# Active avoidance under social conditions recruits the anterior cingulate cortex in male and female rats

**DOI:** 10.21203/rs.3.rs-3750422/v1

**Published:** 2024-01-02

**Authors:** Shannon Ruble, Cassandra Kramer, Lexe West, Karissa Payne, Greg Erickson, Alyssa Scott, Maria Diehl

**Affiliations:** Kansas State University; Kansas State University; Kansas State University; Kansas State University; Kansas State University; Kansas State University; Kansas State University

## Abstract

Actively avoiding potential threats is necessary for survival. Most research has focused on the behavioral and neurobiological processes when individuals avoid potential threats alone, under solitary conditions. Therefore, little is known about how social context affects active avoidance. Using a modified version of the platform-mediated avoidance task, we investigated whether the presence of a social partner attenuates freezing responses and promotes greater avoidance learning compared to avoidance learned under solitary conditions. Rats spent a similar percentage of time avoiding during the conditioned tone under both conditions; however, rats trained under social conditions exhibited greater freezing during the tone and lower pressing for food reward compared to solitary rats. Under solitary conditions, we observed greater avoidance in female compared to male rats, which was not present in rats under social conditions. To gain greater mechanistic insight, we optogenetically inactivated glutamatergic projection neurons in the anterior cingulate cortex (ACC) following avoidance training. Photoinactivation of ACC neurons impaired avoidance expression under social conditions both in the presence and absence of the partner. Under solitary conditions, photoinactivation of ACC delayed avoidance in males but blocked avoidance in females. Our findings suggest that avoidance is mediated by the ACC, regardless of social context, and may be dysfunctional in those suffering from trauma-related disorders. Furthermore, sex differences in prefrontal circuits mediating active avoidance warrant further investigation, given that females experience a higher risk of developing anxiety disorders.

## Introduction

Active avoidance of harm can become maladaptive when it interferes with daily activities. Excessive avoidance is a hallmark feature in those suffering from anxiety disorders [1]. Preclinical studies have investigated the behavioral and neural mechanisms of active avoidance using the platform-mediated avoidance (PMA) task, in which a tone-signaled shock can be avoided by stepping onto a platform, while forgoing sucrose reward [2, 3]. However, prior research using the PMA task has only been conducted when animals learn alone, under solitary conditions, leaving much unknown about how social context may alter avoidance behaviors and their underlying neural mechanisms.

Prior studies on social transmission of fear [4–6] and observational fear conditioning (Allsop et al., 2018; Jeon et al., 2010) and fear extinction [7, 8] have established that fear can be learned from a social partner. Although a few older studies have reported observational learning of active avoidance [9–11], it remains unknown whether avoidance is enhanced in the presence of a social partner.

The prelimbic cortex (PL) is key for avoidance behavior that is acquired during PMA under solitary conditions [12]. PL signals the basolateral amygdala (BLA) and ventral striatum (VS) to bidirectionally control avoidance, allowing the animal to make appropriate decisions while facing competing motivational drives [13]. Studies have demonstrated that the anterior cingulate cortex (ACC) plays a prominent role during social transmission of fear [5] and observational fear learning [14–16]. Therefore, the goals of the current study were twofold: determine the behavioral differences in male and female rats that underwent PMA under social or solitary conditions and determine whether ACC activity is necessary for the expression of avoidance using optogenetic inactivation.

## Materials and Methods

### Subjects

187 Adult Sprague Dawley (n = 93 females, n = 94 males) rats were purchased (Charles River Laboratories, Wilmington, MA) or bred in-house and weighed 280–420 g at the start of experiments. Subjects were same-sex housed in groups of 2–3 rats per cage and maintained on a 12 hr reverse light cycle and handled as previously described [12].

Rats were restricted to 16–18 g/day of standard laboratory rat chow to maintain 85% of their target weight and trained to lever-press for sucrose pellets (BioServ, Flemington, NJ) on a variable interval schedule of reinforcement (VI-30 s). Rats were trained to a criterion of > 10 presses/min for females and > 15 presses/min for males prior to surgical and/or behavioral procedures. All procedures were approved by the Institutional Animal Care and Use Committee of Kansas State University in compliance with the National Institutes of Health guidelines for the care and use of laboratory animals.

### Surgery

For optogenetic experiments, rats were anesthetized under isoflurane and infused with viral vectors in the ACC (+ 1.0 mm AP; ± 0.50 mm ML; 22122.0 mm DV to bregma, at a 0°angle) with 5–6 μL bilaterally (flow rate: 0.05–0.06uL/min). The syringe remained in place for an additional 10 min to reduce backflow. Optical fibers (length, 6 mm; 0.22 NA; 200 nm core from RWD life sciences, Dover, DE) targeted the ACC (+ 1.0 mm AP, ± 3.0 mm ML, −3.0 mm DV, at a 15° angle) and anchored to the skull with cement (C&B-Metabond, Parkell, Brentwood, NY; Ortho Acrylic, Lang Dental, Wheeling, IL). Rats were administered an analgesic (Meloxicam, 1mg/Kg or Flunixin, 1–2 mg/Kg) subcutaneously, and triple antibiotic was applied around the surgical incision. Rats recovered for a minimum of 3 weeks prior to behavioral training to allow for sufficient viral expression (6 weeks before first laser test).

### Behavior

For solitary PMA, rats were trained as previously described [2, 12]. Rats were conditioned with a pure tone (30 s, 4 kHz, 75 dB) co-terminating with a scrambled footshock (2 s, 0.4 mA). The inter-trial interval (ITI) averaged 3 min. An acrylic square platform (14.0 cm each side, 0.33 cm tall) located in the opposite corner of the food dish allowed rats to escape shock. The platform was fixed to the floor and present during all training stages. Rats were trained for 10 days with nine tone-shock pairings per day. A VI-30 schedule was maintained across all training and test sessions.

For social partner PMA, rats were conditioned with the same tone, footshock, and ITI parameters, but with another rat located opposite from a perforated plexiglass barrier in a reconfigured shuttle-box operant chamber which allowed rats access to their own lever, food dish, and platform. Learner Rats were paired with partners that were either previously trained in PMA (Trained Partners) or were naïve to the task (Learner Rat) prior to social partner PMA training. Partners were same-sex and non-cagemates. Rats underwent PMA training with their partner across all daily sessions. After 10 days of social partner PMA, rats underwent an additional session in the absence of their partner on Day 11 (9 tone-shock presentations).

Following 10 days of PMA training, rats in the solitary optogenetic group underwent a test of avoidance expression (2 tones presented without shock), [see 12, 13]. Rats in the social partner optogenetic group underwent two expression tests, one in the presence and one in the absence of their partner. Expression tests were counterbalanced to prevent any order effects.

### Viruses

The adeno-associated viruses (AAVs; serotype 5) were obtained from the University of North Carolina Vector Core (Chapel Hill, NC). Viral titers were 4 × 10^12^ particles/mL for archaerhodopsin (AAV5:CaMKIIα::eArchT3.0-eYFP), and 3 × 10^12^ particles/mL for enhanced yellow fluorescent protein (eYFP) control (AAV5:CaMKIIα::eYFP). Rats expressing eYFP control were used to control for changes due to laser-induced heating of tissue [17]. The CaMKIIα promoter was used to enable transgene expression favoring pyramidal neurons [18] in cortical regions [19–21] (Jones et al., 1994; Van der Oever et al., 2013; Warthen et al., 2016). Viruses were housed in a −80°C freezer until the day of infusion.

### Laser delivery

ACC neurons were bilaterally illuminated using a DPSS green laser (532 nm, constant, 10–12 mW at the optical fiber tip; OptoEngine, Midvale, UT). The laser was activated at tone onset during Tone 1 of the test and persisted throughout the 30 s tone presentation. Laser light passed through a shutter/coupler (200 nm, SRS, Stanford, CA), patch cord (200 nm core, ThorLabs, Newton NJ), rotary joint (200 nm core, 1×2, Doric Lenses, Quebec city, Canada), dual patch cord (0.22 NA, 200 nm core, ThorLabs or RWD life sciences), and optical fibers targeting ACC. Rats were familiarized with dummy patch cords prior to tests.

### Open field task

Locomotion was automatically assessed (ANY-Maze, Stoelting Co, Wood Dale, IL) in an open field arena (90 cm diameter) during 30 s laser off and 30 s laser on time periods. A 6 min acclimation period preceded laser illumination. Speed and distance traveled were used to assess locomotion, and time in center was used to assess anxiety.

### Pressing test

Lever-pressing was assessed with a VI-30 schedule and began with a 60 s acclimation period, followed by 7 Laser On (30 s) trials and 6 60 s Laser Off (60 s) intervals. The number of lever presses was compared during Laser Off and Laser On periods within subjects using a paired-t-test.

### Histology

After experiments, rats were deeply anesthetized with sodium pentobarbital (450 mg/kg i.p.) and transcardially perfused with 0.9% saline followed by a 10% buffered formaldehyde. Brains were removed and stored in 30% sucrose for cryoprotection for at least 72 h before sectioning. Coronal sections were cut (40 μm), mounted on slides and analyzed for viral expression and optical fiber placement.

### Data Collection and Analysis

Behavior was recorded with digital video cameras and quantified using ANY-Maze software (Stoelting, Wood Dale, IL). The number of shocks avoided was calculated as the rat spending at least 1.75 s on the platform during the 2 s shock period of each tone presentation. Shock reactivity was calculated using the maximum speed of each rat during exposure to the first shock on Day 1 of PMA.

Multilevel regressions were performed to assess significant differences in several behaviors observed during PMA. For percentage of time spent on the platform and time spent freezing during the tone (with the maximum time, 30 seconds, included as the model weight parameter), multilevel binomial logistic regressions were performed to account for individual differences and proportion data being bounded by zero and one [22]. Model parameter estimates are available in Supplementary Tables S1 (freezing) and S2 (platform time). For number of shocks avoided and number of ITI presses (30 s prior to tone onset), multilevel negative binomial regressions were performed to account for individual differences and positively skewed count data. Model parameter estimates are available in Supplementary Tables S3 (lever presses) and S4 (shocks avoided). All models included Group Type (social vs solitary), Sex, and Session (day of training) as fixed effects and Individual and Tone Number as random effects. To investigate effects of Partner Type (Learner Rat vs. Trained Partner), additional models were run only on the social partner PMA data, with Partner Type added as a predictor variable instead of Group Type. Parameter estimates for these models are available in Supplementary Tables S1-S8.

**Figure 1.**
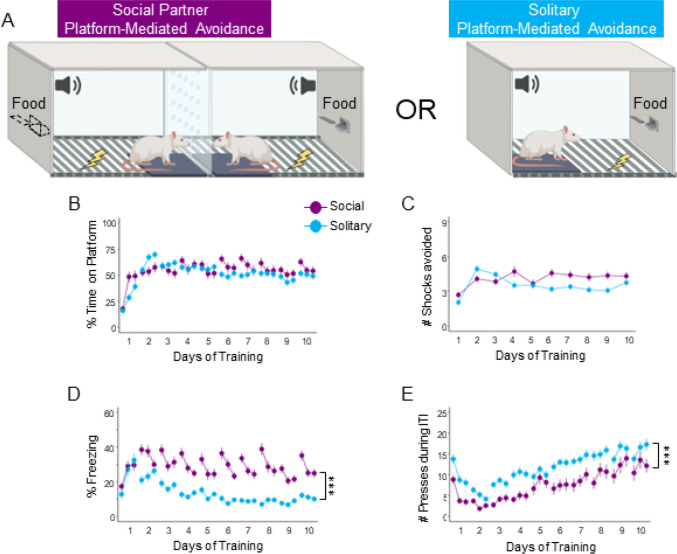
Platform-mediated avoidance (PMA) under social conditions increases freezing and decreases pressing during the intertrial interval (ITI) compared to PMA under solitary conditions. **A**. Schematic of PMA under social (left, purple, n=42) and solitary (blue, n=59) conditions. **B**. Percentage time spent on the platform during the tone. **C**. Number of shocks avoided per day. There was no significant difference in time on platform (multilevel binomial logistic regression, *z*=−1.252, *p*=0.211) or number of shocks avoided (multilevel negative binomial regression, *z*=−0.552, p=0.581) between rats trained under social compared to solitary conditions. **D**. Percentage of time freezing during the tone. **E**. Number of lever presses during the ITI period. Rats trained under social conditions showed a significant increase in freezing (multilevel binomial logistic regression, *z*=4.068, *p*<0.0001) and a significant decrease in ITI pressing (multilevel negative binomial regression, *z*=−6.034, *p*<0.0001) compared to rats trained under solitary conditions. Data shown across 10 days of training (trials shown in blocks of 3) for rats trained in PMA under social or solitary conditions. Data are shown as mean ± SEM; ***p<0.001.

**Figure 2.**
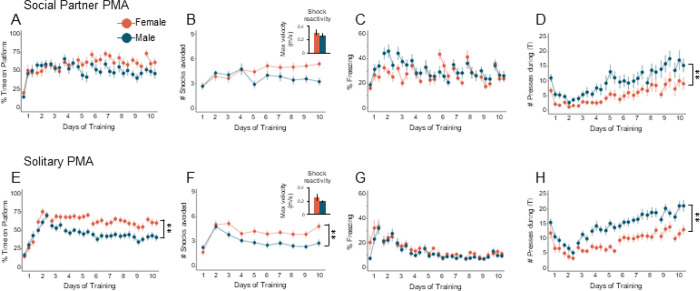
PMA under social conditions reduces behavioral sex differences that are observed during PMA under solitary conditions. **A**. Percentage of time on platform during the tone, **B**. Number of shocks avoided and shock reactivity (inset), as measured by the maximum velocity of each rat during Tone 1 on the first day of training. **C**. Percentage of freezing during the tone, and **D**. Number of lever presses during the ITI in female (n=21, salmon) and male (n=21, teal) rats trained under social conditions. During social partner PMA training, female rats pressed significantly less than male rats (multilevel negative binomial regression, with Tukey’s post hoc contrast tests, *z*=−3.112, *p*=0.0019), but there were no sex differences in time on platform (multilevel binomial logistic regression, with Tukey’s post hoc contrast tests, z=1.308, *p*=0.191), number of shocks avoided (multilevel negative binomial regression, with Tukey’s post hoc contrast tests, *z*=1.590, p=0.112), or freezing (multilevel binomial logistic regression, with Tukey’s post hoc contrast tests, *z*=−0.673, *p*=0.501). **E**. Percentage of time on platform during the tone, **F**. Number of shocks avoided and shock reactivity (inset), **G**. Percentage of freezing during the tone, and **H**. Number of presses during the ITI in female (n=27, salmon) and male (n=32, teal) rats trained under solitary conditions. During solitary PMA training, female rats spent significantly more time on the platform (multilevel binomial logistic regression, with Tukey’s post hoc contrast tests, *z*=3.174, *p*=0.0015), avoided significantly more shocks (multilevel negative binomial regression, with Tukey’s post hoc contrast tests, *z*=2.949, *p*=0.0032), and pressed significantly less (multilevel negative binomial regression, with Tukey’s post hoc contrast tests, *z*=−3.044, *p*=0.0023) compared to male rats. Data shown across 10 days of training (trials shown in blocks of 3). Data are shown as mean ± SEM; **p<0.01.

**Figure 3.**
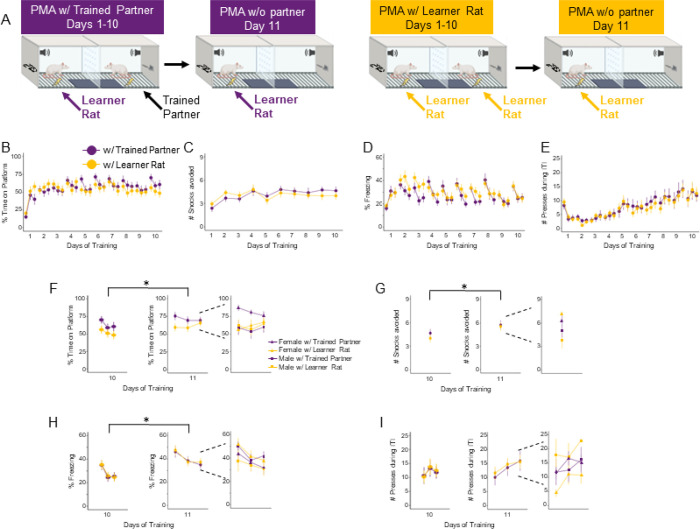
Avoidance and freezing increase in the absence of a social partner. **A.** Avoidance and freezing increase in the absence of a social partner. A. Schematic of PMA under social conditions with a Trained Partner (n=20, purple) or another Learner Rat (n=22, yellow). **B**. Percentage of time on platform, C. Number of shocks avoided, **D**.Percentage of freezing, and **E**. Pressing during the ITI across the 10 days of training. Rats showed similar levels of avoidance (time on platform: multilevel binomial logistic regression, *z*=−1.548, *p*=0.122; number of shocks avoided: multilevel negative binomial regression, z=0.391, p=0.695), freezing (multilevel binomial logistic regression, *z*=−1.168, *p*=0.243), and ITI pressing (negative binomial regression, *z*=0.218, *p*=0.828). **F**. Percentage of time on platform, **G**. Number of shocks avoided, **H**.Percentage of freezing, and **I**. Pressing during the ITI on training Day 10 (left) and Day 11 in the absence of the partner (middle), and Day 11 data split by partner type and sex. On Day 11, Learner Rats trained with either partner type showed increased time on platform (multilevel binomial logistic regression, with Trained Partner: z = −31.104, p < 0.0001; with Learner Rat: z = −7.669, p < 0.0001); greater # of shocks avoided (multilevel negative binomial regression, with Trained Partner: z = −3.455, p = 0.0005; with Learner Rat: z = −2.519, p = 0.0118), and increased freezing with trained with a Trained Partner (multilevel binomial logistic regression, z = −3.921, p < 0.0001). Data are shown in blocks of 3 for each day, mean ± SEM. *p<0.05.

All analyses for experiments in Figs. 1–3 were conducted in R (version 4.2.1), using the lme4 library, version 1.1–30 [23]. The emmeans library, version 1.8.0 [24] was used to calculate post-hoc Tukey tests and estimated marginal means from each model (all reported means model estimates).

For optogenetic manipulations, repeated-measures ANOVA, followed by post-hoc Tukey analyses, or Student’s two-tailed t tests were used where appropriate using Prism (Graphpad, La Jolla, CA), or JMP (SAS, Cary, NC) software. Aspects of operant box schematic were created with Biorender.com.

## Results

### Platform-mediated avoidance (PMA) under social conditions increases freezing and decreases pressing compared to PMA under solitary conditions.

Previous studies of PMA under solitary conditions have shown that freezing decreases and avoidance increases as rats progress through training [2, 25]. To investigate how the presence of another rat can affect avoidance, several behaviors were compared across PMA training under social or solitary conditions. One group of rats was trained in social partner PMA, in which rats underwent training simultaneously with another rat (Fig. 1A, left) and another group of rats was trained in solitary PMA, in which they learned avoidance alone (Fig. 1A, right). Both groups underwent training for 10 days as previously described [2, 12]. We utilized multilevel regressions to describe behavior observed during PMA under solitary or social conditions (see Methods and Supplemental Information on regression models and parameter estimates). Across 10 days of training, both social (purple) and solitary (blue) groups showed similar levels of avoidance, as measured by the percentage of time spent on the platform during the tone (Fig. 1B) and the average number of shocks avoided on each day (Fig. 1C). There was no significant effect of Group Type for time on platform (social vs. solitary; *z* = −1.252, *p* = 0.211). or number of shocks avoided (*z* = −0.552, *p* = 0.581).

Interestingly, rats trained under social conditions showed greater freezing compared to rats trained under solitary conditions (Fig. 1D). A multilevel regression showed a significant effect of Group Type predicting time freezing (*z* = 4.068, *p* < 0.0001), with social rats freezing more than solitary rats. In addition, solitary rats pressed significantly more than social rats (*z* = −6.304, p < 0.0001; Fig. 1E). Therefore, PMA training under social conditions enhanced freezing responses while decreasing food-seeking, with little effect on avoidance, compared to PMA training under solitary conditions.

### Solitary PMA reveals sex differences that are not present during social partner PMA.

Most PMA studies have used only male rats [2, 12, 13, 25–28]. However, recent studies have included both sexes [29–31]. To investigate sex differences in PMA under social or solitary conditions, we included both male and female rats in all experiments. Data in Fig. 2A-H is the same data as in Fig. 1 but separated by males (teal) and females (salmon). Post-hoc Tukey tests on the previous regression models showed that males and females under social conditions showed no significant differences in avoidance (Fig. 1A; *z* = 1.308, *p* = 0.191), number of shocks avoided (Fig. 2B; z = 1.590, *p* = 0.112), or freezing (Fig. 2C; *z* = −0.673, *p* = 0.501). However, males showed significantly increased pressing compared to females (Fig. 2D; *z* = −3.112, *p* = 0.0019).

During solitary PMA, females avoided significantly more than males, as measured by time on platform (Fig. 2E; *z* = 3.174, *p* = 0.0015;) and number of shocks avoided (Fig. 2F; *z* = 2.949, *p* = 0.0032). This effect was not due to shock reactivity (Fig. 2F, inset, t-test, t_(47)_ = 1.465, p = 0.150). Post-hoc Tukey tests on the regression model showed no significant differences in freezing between males and females (Fig. 2G, z = 1.903, *p* = 0.0571), but males pressed more than females (Fig. 2H, z = −3.044, *p* = 0.0023). Altogether, these results suggest that sex differences are suppressed during social partner PMA compared to solitary PMA.

The significant effects of Group Type (social vs. social) suggests that there are differences between groups in freezing and pressing regardless of sex differences. To confirm this, we directly compared behaviors in females between Group Type and males between Group Type using contrast tests on the multilevel regression models. Post-hoc Tukey tests identified significant differences in freezing between social and solitary females (*z* = 4.77, *p* < 0.0001) and between social and solitary males (*z* = 7.449, *p* < 0.0001; Supplementary Fig. 1C and G). Similarly, significant differences were found in pressing between social and solitary females (*z* = −4.069, *p* < 0.0001), and between social and solitary males (*z* = −3.623, *p* = 0.0003; Supplementary Fig. 1D and H). Collectively, freezing was enhanced and pressing was diminished during social partner PMA, regardless of sex.

### Previous PMA experience of a social partner does not affect acquisition but alters avoidance and freezing in the absence of the partner.

To determine whether the previous experience of a social partner affects avoidance, rats were paired with same-sex non-cagemate partners that had previously undergone PMA (Learner Rat with a Trained Partner) or were naïve to the task at the start of PMA (Learner Rat with another Learner Rat). We compared the same behaviors (time on platform, number of shocks avoided, time freezing, and pressing) across the 10 days in Learner Rats trained with a Trained Partner (purple) or Learner Rats trained with another Learner Rat (yellow). A multilevel regression found no significant effect of Partner Type on avoidance (Fig. 3B; *z* = −1.548, *p* = 0.122), freezing (Fig. 3D; *z* = −1.168, *p* = 0.243), number of shocks avoided (Fig. 3C; *z* = 0.391, *p* = 0.695), or pressing (Fig. 3E; *z* = 0.218, *p* = 0.828). Altogether, partner types learn social partner PMA in a similar fashion.

We were next interested in whether the absence of the partner would alter behaviors during PMA. We compared the same behavioral measures of avoidance, freezing, and pressing in the presence of their partner (on Day 10) versus in the absence of their partner (on Day 11) using contrast comparisons on the previous multilevel regression models. Learner Rats with either Partner type spent significantly more time on the platform on Day 11 than Day 10 (Fig. 3F, Trained Partner *z* = −31.104, *p* < 0.0001; Learner Rat *z* = −7.669, *p* < 0.0001). Learner rats trained with either Partner Type avoided more shocks on Day 11 than Day 10 (Fig. 3G, Trained Partner *z* = −3.455, *p* = 0.0005; Learner Rat *z* = −2.519, *p* = 0.0118).

There was also a significant increase in freezing from Day 10 to Day 11 for Learner Rats with a Trained Partner (Fig. 3H, z = −3.921, *p* < 0.0001). There were no significant differences in freezing between groups on Day 11 (with a Learner rat: *z* = −0.158; *p* = 0.874; with a Trained Partner *z* = 1.172; p = 0.241). There were small significant differences in pressing between Day 10 and 11 for Learner Rats with either Partner Type (Fig. 3I; Trained Partner *z* = −15.022, *p* < .0001; Learner Rat Partner *z* = −17.218, *p* < .0001) but no significant differences in pressing between groups on Day 11 (with a Learner rat: *z* = −1.423; *p* = 0.1547; with a Trained Partner: *z* = −1.065; *p* = 0.2871).

We next assessed whether there were any sex differences (regardless of Partner Type) that depended on partner presence. Both males and females spent significantly more time on the platform on Day 11 than Day 10, and females avoided significantly more than males when their partner was absent (Day 11), regardless of Partner Type during training (Supplementary Fig. 2A). Females also avoided significantly more shocks (Supplementary Fig. 2B) and showed a small significant increase in freezing for females from Day 10 to 11 (Supplementary Fig. 2C). Taken together, partner absence augments fear and avoidance responses, especially in females, when avoidance is learned socially.

### Photoinactivation of ACC impairs avoidance under social conditions.

**Figure 4.**
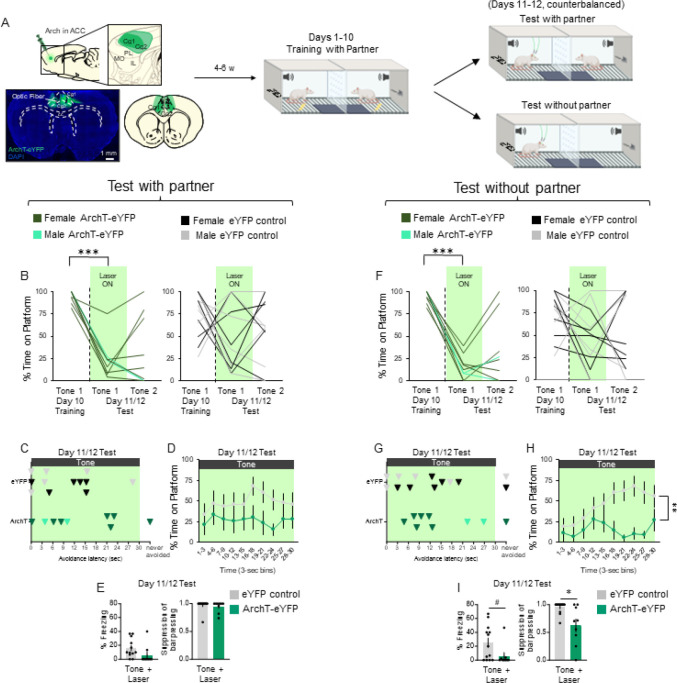
Photoinactivation of ACC impairs avoidance under social conditions regardless of partner presence. **A.** Schematic of virus infusion, location of min/max expression of AAV, followed by avoidance training and tests. At Test w/ partner and w/o partner (Days 11/12), 532nm light was delivered to ACC during the entire 30-second tone presentation (Tone 1). **B**. Percentage of time on platform at Cond (Day 10, Tone 1) and Test (Days 11/12, Tone 1 with laser ON and Tone 2 with laser OFF) for ArchT-eYFP rats (n=9, green) and eYFP-control (n=12–13, grey) when the partner was present. Avoidance decreased in ArchT rats (repeated measures ANOVA, main effect of trial (F_(2,38)_=13.88, p<.001) and interaction between trial and AAV (F_(2,38)_=3.70, p=0.034); post hoc Tukey test revealed significant decrease between Tone 1 of Day 10 vs. Day 11 in ArchT-eYFP (p=0.002), but not in eYFP controls (p=0.478)). **C**. Latency of avoidance for each rat during Test w/ partner (Tone 1 at Test; Male eYFP control, grey; Female eYFP control, black; Male ArchT, light green, Female ArchT, dark green). D. Percentage of time on platform in 3 s bins (Tone 1 at Test) revealed no effect of photoinactivating ACC neurons compared to eYFP controls in the presence of the partner (repeated measures ANOVA, post hoc Tukey, F_(9,126)_= 0.891, p=0.535). **E**. Percentage of freezing (left) and suppression of bar pressing (right) during the Tone+Laser trial. F. Percentage time on platform at Cond (Day 10, Tone 1) and Test (Day 11, Tone 1 with laser ON and Tone 2 with laser OFF) for eYFP-control (n=10, grey) and ArchT-eYFP rats (n=7, green) when the partner was absent. Avoidance decreased in ArchT rats (repeated measures ANOVA, main effect of trial (F_(2,40)_=21.79, p<.001) and interaction between trial and AAV (F(2,40)=4.21, p=.022); post hoc Tukey test revealed significant decrease between Tone 1 of Day 10 vs. Day 11 in ArchT-eYFP (p<0.001), but not in eYFP controls (p=0.098)). **G**. Latency of avoidance for each rat during Test w/o partner (Tone 1 at Test; Male eYFP control, grey; Female eYFP control, black; Male ArchT-eYFP, light green, Female ArchT-eYFP, dark green). **H**. Percentage of time on platform in 3 s bins (Tone 1 at Test) revealed a significant reduction in avoidance when silencing ACC neurons compared to eYFP controls in the absence of the partner (repeated measures ANOVA, post hoc Tukey, F_(9,135)_=3.09, p=0.002). **I**. Percentage of freezing (left) and suppression of bar pressing (right) during the Tone+Laser trial revealed a trend toward less freezing in ArchT rats (t_(20)_=2.036, p=0.0552) and significantly lower suppression of bar pressing in ArchT rats (t_(20)_=3.225, p=0.0042). All data are shown as mean ± SEM; #p<0.06, *p<0.05, **p<0.01, ***p<0.001.

Previous studies have linked ACC activity with social learning [15, 16, 32]. We therefore reasoned that ACC activity would be necessary for social partner PMA. To assess this, we used an optogenetic approach to test if inactivation of ACC neurons would impair avoidance under social conditions. ArchT-eYFP (AAV5:CaMKIIa::eArchT3.0-eYFP) or eYFP control was expressed in ACC gluatamatergic projection neurons [18–21]. Following viral infusion, surgical placement of optical probes, and a 4–5 week period to allow viral expression, rats were trained in social partner PMA (Fig. 4A). Histological analysis confirmed that expression of ArchT-eYFP was largely confined to the ACC (including anatomical areas Cg1 and Cg2) with minimal spread to prelimbic cortex (PL; Fig. 4A, bottom left).

Following 10 days of social partner PMA (Fig. 4A, middle), Learner rats underwent two avoidance expression tests: one in the presence and another in the absence of their partner (Fig. 4A, top and bottom right, respectively). Green laser was presented concurrently with the first 30 s tone (Laser ON), but not during Tone 2 (Laser OFF). A 2-way repeated measures ANOVA comparing time on platform in ArchT-eYFP and eYFP controls during Tone 1 on the last day of training, and Tones 1 (Laser ON) and 2 (Laser OFF) of test with the partner present revealed a significant main effect of trial (F_(2,38)_ = 13.88, p < .001) and interaction between trial and AAV (F_(2,38)_ = 3.70, p = 0.034), but not a significant main effect of AAV (F_(1,19)_ = 1.81, p = 0.194; data collapsed across partner type and sex). Post-hoc Tukey tests revealed a significant decrease in time on platform between Tone 1 on the last day of training and Tone 1 of test in the ArchT-eYFP group (p = 0.002), but not in the eYFP control group (p = 0.478; Fig. 4B). When comparing avoidance latency across the tone + laser trial between ArchT-eYFP and eYFP control rats, there was no significant difference (Fig. 4C, t_(19)_ = 1.122, p = 0.276), nor across the timecourse of avoidance, as measured in 3 s bins of the tone period (Fig. 4D, repeated measures ANOVA: F_(9,126)_ = 0.891, p = 0.535). Finally, photoinactivation had no effect on freezing (t_(19)_ = 1.683, p = 0.109) nor on suppression of bar pressing (t_(19)_ = 0.607, p = 0.551) in the presence of the partner (Fig. 4E).

In the absence of the partner, a 2-way repeated measures ANOVA comparing time on the platform in ArchT-eYFP and eYFP controls during Tone 1 of the last day of training, and Tones 1 and 2 of test revealed a significant main effect of trial (F_(2,40)_ = 21.79, p < .001) and interaction between trial and AAV (F_(2,40)_ = 4.21, p = .022), but not a significant main effect of AAV (F_(1,20)_ = 1.01, p = 0.328). Post-hoc Tukey tests revealed a significant decrease in time on the platform between Tone 1 on the last day of training and Tone 1 of test in the ArchT-eYFP group (p < .001), but not in the eYFP control group (p = 0.098; Fig. 4F). Photoinactivation had no effect on avoidance latency (Fig. 4G, t_(20)_ = 0.539, p = 0.596). When comparing the timecourse of avoidance between ArchT-eYFP and eYFP control rats, photoinactivation significantly reduced avoidance throughout the tone (Fig. 4H, F_(9,135)_ = 3.09, p = 0.002)). Finally, photoinactivation had no effect on freezing (t_(20)_ = 2.036, p = 0.0552) but a significant decrease on suppression of bar pressing (t_(20)_ = 3.225, p = 0.0042) in absence of the partner (Fig. 4I). Altogether, photoinactivation of ACC somata during the tone impaired avoidance expression during the presence and absence of the partner.

### Photoinactivation of ACC delays avoidance under solitary conditions in males but blocks avoidance in females.

**Figure 5.**
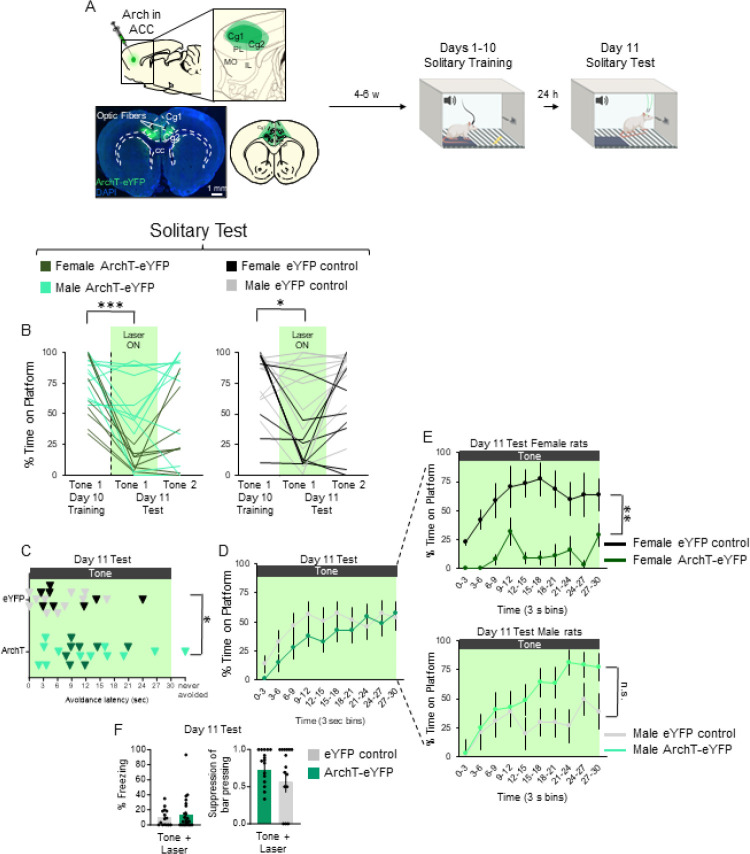
Photoinactivation of ACC projection neurons delays avoidance expression in male rats but blocks avoidance expression in female rats under solitary conditions. **A.** Schematic of virus infusion, location of min/max expression of AAV, followed by avoidance training and test. At Test, 532nm light was delivered to ACC during the entire 30-second tone presentation (Tone 1). **B**. *Left*: Percentage of time on platform at Cond (Day 10, Tone 1) and Test (Day 11, Tone 1 with laser ON and Tone 2 with laser OFF) for ArchT-eYFP rats (n=20, right) and eYFP control (n=15, left). Avoidance was impaired in ArchT-eYFP rats (repeated measures ANOVA, main effect of trial (F_(2,66)_=16.53, p<0.001); post hoc Tukey test revealed a significant decrease between Tone 1 of Day 10 vs. Day 11 in ArchT-eYFP (p<0.001), but not in eYFP controls (p=0.478)). **C.** Latency of avoidance for each rat (Tone 1 at Test). ArchT-eYFP rats showed a trend toward a delay in avoidance latency in the ArchT-eYFP rats (t_(33)_=2.033, p=0.0502). **D**. Percentage of time on platform in 3 s bins (Tone 1 at Test) revealed no significant differences between groups (repeated measures ANOVA, post hoc Tukey, F_(9,279)_=1.25, p=0.265). *Insets*: Percent freezing (left) and suppression of bar pressing (right) during the Tone+Laser trial. **E**. Percentage of freezing (left) and suppression of bar pressing (right) during the Tone+Laser trial. **F**. Percentage of time on platform in 3 s bins (Tone 1 at Test) revealed a significant delay in avoidance when silencing ArchT-eYFP neurons in male rats (light green) while blocking avoidance in female rats (dark green; repeated measures ANOVA F_(9,162)_=3.76, p<0.001, post hoc Tukeys, all p’s<0.001 15–30 sec). Data shown as mean ± SEM; ***p<0.001.

To determine whether ACC activity is necessary for avoidance under solitary conditions, we photoinactivated these neurons during an expression test following solitary PMA. Similar to the experiment in Fig. 4, rats were infused with either ArchT-eYFP or eYFP control and subsequently underwent solitary PMA (Fig. 5A). A 2-way repeated measures ANOVA comparing time on the platform in ArchT-eYFP and eYFP controls during Tone 1 of the last day of training, and Tones 1 and 2 of test showed a significant main effect of trial (F_(2,70)_ = 18.49, p < 0.001), but no main effect of AAV (F_(1,35)_ = 2.83, p = 0.102), or their interaction (F_(2,70)_ = 0.555, p = 0.577; Fig. 5B). Post-hoc Tukey tests revealed a significant decrease in time on the platform between Tone 1 on the last day of training and Tone 1 of test in the ArchT-eYFP group (p < .0.001) but also in the eYFP control group (p = 0.004). ArchT-eYFP rats showed a significantly longer avoidance latency (mean: 12.3 s) compared to eYFP controls (mean: 7.34 s, t_(37)_ = 2.132, p = 0.0397, Fig. 5C). Analysis of the timecourse of avoidance showed no differences between ArchT-eYFP and eYFP control rats (Fig. 5D, repeated measures ANOVA, F_(9,279)_ = 1.25, p = 0.265). However, female ArchT-eYFP rats showed significantly less avoidance compared to female eYFP controls (p = 0.0010), whereas male ArchT-eYFP and male eYFP control rats did not show any significant differences (p = 0.189, Fig. 5E). Interestingly, ArchT-eYFP males showed a delay in avoidance compared to ArchT-eYFP females, who showed a near complete impairment of avoidance across the 30 s tone (comparing light and dark green lines across both graphs in Fig. 5E; repeated measures ANOVA, F_(9,162)_ = 3.76, *p* < 0.001, post hoc Tukeys, all p’s < 0.001 after 15 s). Photoinactivation had no effect on freezing (t_(37)_ = 0.607, p = 0.548) or suppression of bar pressing (t_(31)_ = 1.023, p = 0.314; Fig. 5F). Thus, photoinactivation of ACC somata blocked avoidance in females, but delayed avoidance in males.

Finally, photoinactivation had no effect on spontaneous bar-pressing between ArchT-eYFP and eYFP controls (t-test, t_(24)_ = 0.343, p = 0.738; ArchT-eYFP n = 11, M = 4.7, eYFP n = 13, M = 4.8). Photoinactivation also had no effect on locomotion in an open field (t_(41)_ = −1.22, p = 0.232; ArchT-eYFP n = 17, M = 1.1 m, eYFP n = 24,M = 0.97 m), nor on anxiety levels, as both groups spent similar amounts of time in the center of the open field (t_(41)_ = −1.35, p = 0.184; ArchT-eYFP n = 17, M = 3.3 s, eYFP n = 24, M = 2.0 s).

## Discussion

We developed a behavioral task to assess the acquisition of PMA under social conditions and compared learning between male and female rats trained in PMA under social or solitary conditions. We found that females avoided more than males, whereas males pressed more for sucrose reward than females during PMA training, and this persisted, even in the absence of the social partner. Sex differences were more prominent in solitary vs. social rats. Thus, a social context of aversive learning appears to suppress sex differences, causing female rats to “act more like” male rats. We also found that, regardless of sex, freezing was enhanced under social vs. solitary conditions. In addition, when rats underwent social partner PMA, the prior experience of their partner did not affect behavior during training. However, removing their partner increased avoidance in females and freezing in both sexes, especially if their partner had previous PMA experience. We next found that ACC activity was necessary for the expression of avoidance in rats that learned under social and solitary conditions, but in different ways. In social partner PMA, ACC activity was necessary for the expression of avoidance both in the presence and absence of the partner. In solitary PMA, ACC activity modulated avoidance expression in a sex-dependent manner, such that photoinactivating ACC had a milder effect of delaying avoidance in males, but a stronger effect of blocking avoidance in females. Below, we highlight some unexpected findings from our study along with possible explanations and future research directions.

Our surprising finding that freezing increased during social partner PMA compared to solitary PMA (Fig. 1C) suggests that Learner Rats associated their partner with danger. This agrees with studies showing that rats can associate another rat with a shock US [33]. A rat that is freezing to a CS can also become a signal for danger, in addition to the CS itself [34]. It is also possible that Learner Rats increased freezing during social partner PMA due to detecting lack of movement from their partner, similar to previous studies showing that social transmission of fear can be propagated by silence caused by cessation of movement [35]. In the current study, it is likely that Learner Rats perceived their partner as a negative stimulus rather than a positive stimulus that would have buffering fear responses. This agrees with studies showing that partner presence *during* a traumatic event does not reduce fear responses, but rather a partner present *after* a traumatic event does reduce fear responses [8]. Future studies using social partner PMA can address this question by testing whether presenting a partner after PMA training (under solitary conditions) would decrease freezing and facilitate extinction of avoidance.

We also observed lower press rates under social compared to solitary conditions, suggesting that Learner Rats may be less motivated for food reward when another rat is present. This may be due to Learner Rats investigating their partner to obtain sensory information about PMA. Studies have found that during social learning, rodents use auditory and visual cues [36] as well as olfactory cues [37, 38] to gather information about the environment. In the current study, Learner Rats may observe their partner avoid, causing them to also avoid or hear an alarm vocalization emitted by their partner during the tone, but these were not measured. Future studies should aim to investigate whether social partner PMA relies on visual, auditory, or olfactory cues between partners.

Studies investigating social interactions and social learning have pointed to the anterior cingulate cortex (ACC) as a key region across many species [16]. Previous research suggests that ACC is necessary for the integration of social cues and information about aversive stimuli, which are mediated by connections between the ACC and basolateral amygdala (BLA) [39]. The ACC is a highly interconnected cortical brain region, projecting densely to the PL [40, 41] and BLA [42–44]. The ACC may signal to the PL information about the social partner during PMA. The ACC also comprises part of the value processing network together with the BLA, which processes observed emotion and social interactions [16, 32]. ACC neurons may signal the BLA during PMA under social conditions, thereby allowing Learner rats to use social information to avoid danger.

We found that during PMA under solitary conditions, ACC photoinactivation had a mild effect of delaying avoidance in males but had a stronger effect of blocking avoidance in female, suggesting that ACC is recruited differentially in male and female rats. This suggests that female avoidance circuits rely more heavily on ACC, whereas male avoidance circuits may rely more on other nodes of the avoidance circuit such as PL [12, 13]. Future electrophysiological studies could determine ACC correlates of behavior during PMA and how ACC activity may differ under social partner vs. solitary PMA. For example, Partner Type may alter ACC activity during social partner PMA, similar to studies showing that dominance status changes neuronal activity during social interactions [45].

Excessive avoidance is a key symptom in several neuropsychiatric illnesses including PTSD [46, 47], OCD [48], social anxiety disorder (SAD) [49], depression [50], and autism spectrum disorder (ASD) [51]. The human homolog of the rat ACC, Brodmann areas 24a/b [16], has been implicated in these disorders [OCD: 52, 53], [SAD: 54], [depression: 55], [ASD: 56]. Interestingly, a meta-analysis reported that the ACC was one of seven regions with disrupted functional connectivity across different anxiety disorders [57]. Therefore, findings of the current study are consistent with clinical studies that the ACC is an area of interest for developing treatments to resolve symptoms of these neuropsychiatric disorders.

The PMA task has previously been used as a model to study extinction-based treatment for PTSD and OCD [25, 26]. Therefore, modifying the PMA task to include a social context could be used as an animal model of behavior to study other anxiety disorders such as SAD, in which social context is a key factor. Finally, our findings that females avoided more than males and showed a stronger impairment of avoidance when ACC was optogenetically inactivated suggest that ACC may be a key factor in understanding why females are at a greater risk of an anxiety disorder.
