# Active avoidance recruits the anterior cingulate cortex regardless of social context in male and female rats

**DOI:** 10.21203/rs.3.rs-3750422/v2

**Published:** 2024-06-07

**Authors:** Shannon Ruble, Cassandra Kramer, Lexe West, Karissa Payne, Halle Ness, Greg Erickson, Alyssa Scott, Maria M. Diehl

**Affiliations:** Department of Psychological Sciences, Kansas State University, Manhattan, KS 66506

**Keywords:** prefrontal cortex, fear, sex differences, optogenetics

## Abstract

Actively avoiding danger is necessary for survival. Most research has focused on the behavioral and neurobiological processes when individuals avoid danger alone, under solitary conditions. Therefore, little is known about how social context affects active avoidance. Using a modified version of the platform-mediated avoidance task in rats, we investigated whether the presence of a social partner attenuates conditioned freezing and enhances avoidance learning compared to avoidance learned under solitary conditions. Rats spent a similar percentage of time avoiding during the tone under both conditions; however, rats trained under social conditions exhibited greater freezing during the tone as well as lower rates of darting and food seeking compared to solitary rats. Under solitary conditions, we observed higher levels of avoidance in females compared to males, which was not present in rats trained under social conditions. To gain greater mechanistic insight, we optogenetically inactivated glutamatergic projection neurons in the anterior cingulate cortex (ACC) following avoidance training. Photoinactivation of ACC neurons reduced expression of avoidance under social conditions both in the presence and absence of the partner. Under solitary conditions, photoinactivation of ACC delayed avoidance in males but blocked avoidance in females. Our findings suggest that avoidance is mediated by the ACC, regardless of social context, and may be dysfunctional in those suffering from trauma-related disorders. Furthermore, sex differences in prefrontal circuits mediating active avoidance warrant further investigation, given that females experience a higher risk of developing anxiety disorders.

## 1. Introduction

The ability to assess danger and respond appropriately is critical for an individual’s well-being. Active avoidance is a commonly used strategy to appropriately evade danger but can become maladaptive when avoidance is excessive and impedes the ability to complete daily activities. Excessive avoidance is a hallmark feature in those suffering from anxiety disorders (American Psychiatric Association, 2013). Preclinical studies have investigated the behavioral and neural mechanisms of active avoidance using the platform-mediated avoidance (PMA) task. During this task, rats are trained to avoid a tone-signaled shock by stepping onto a safe platform, which comes at the cost of lever-pressing for a sucrose reward (Bravo-Rivera et al., 2014; Diehl et al., 2019). However, prior research using the PMA task has only been conducted when animals learn alone, under solitary conditions, leaving much unknown about how social context may alter avoidance behaviors and their underlying neural mechanisms.

Previous rodent studies have focused on learning about danger through a social partner using various tasks. During social transmission of conditioned fear, a naïve rat that interacts with a fear-conditioned cagemate will subsequently express greater freezing to the conditioned stimulus compared to another naïve rat that did not interact with a fear-conditioned cagemate (Brandl et al., 2022; Bruchey et al., 2010; Jones & Monfils, 2016). Learning about danger through a social partner has also been demonstrated using observational learning. During observational fear conditioning, an observer rodent witnesses a demonstrator rodent undergo fear conditioning and is later tested for the same fear responses. Enhanced fear learning via observation has been reported during fear expression (Allsop et al., 2018; Jeon et al., 2010), fear extinction (Brill-Maoz & Maroun, 2016; Gorkiewicz et al., 2023), and expression of shuttle avoidance (Del Russo, 1975; John et al., 1968; Presley & Riopelle, 1959). Altogether, these studies show that fear and avoidance can be learned from a social partner; however, it remains unknown whether avoidance learning is enhanced in the presence of a social partner when rodents learn simultaneously.

The neural circuits regulating active avoidance have been previously characterized in male subjects that learn alone (for review, see Diehl et al., 2019). The prelimbic cortex (PL) is key for avoidance behavior in PMA under solitary conditions (Diehl et al., 2018). PL signals both the basolateral amygdala (BLA) and the ventral striatum (VS) to bidirectionally control avoidance, allowing the animal to make appropriate decisions while facing competing motivational drives (Diehl et al., 2020). Prior studies have reported that the anterior cingulate cortex (ACC) plays a key role for social transmission of fear and observational fear conditioning (for review, see Burgos-Robles et al., 2019; Debiec & Olsson, 2017; Olsson & Phelps, 2007). Despite these recent advances uncovering the neural substrates of social fear learning, there are no studies investigating the neural substrates of active avoidance acquired under social conditions.

The current study had two main goals. First, we were interested in whether avoidance learning differs between male and female rats under social vs. solitary conditions. To do this, we modified the PMA task so that two rats could perform the task together while maintaining access to their own lever, food dish, and platform. Second, we were interested in whether activity in the ACC is necessary for the expression of active avoidance under social conditions. To test this, we optogenetically inactivated ACC neural activity during the tone of the PMA task.

## 2. Materials and Methods

### 2.1 Subjects

187 Adult male and female Sprague Dawley (n= 93 females, n=94 males from 26 litters) rats were bred in-house from rats purchased from a commercial vendor (Charles River Laboratories, Wilmington, MA) and were 3–5 months old, weighing 280–420 g at the start of experiments. Subjects were same-sex housed in groups of 2–3 rats per cage and maintained on a 12 hr reverse light cycle (lights off at 0830 hrs) and handled as previously described (Diehl et al., 2018). All experiments were completed between 0900–1800 hrs during the active dark cycle of the rats.

Rats were restricted to 16–18 g/day of standard laboratory rat chow to maintain 85% of their target weight and trained to lever-press for sucrose pellets (BioServ, Flemington, NJ) on a variable interval schedule of reinforcement (VI-30 s). Rats were trained to a criterion of >10 presses/min for females and >15 presses/min for males prior to surgical and/or behavioral procedures. All procedures were approved by the Institutional Animal Care and Use Committee of Kansas State University in compliance with the National Institutes of Health guidelines for the care and use of laboratory animals.

### 2.2 Surgery

For optogenetic experiments, rats were anesthetized under isoflurane and infused with viral vectors in the ACC (+1.0 mm AP; ± 0.50 mm ML; −2.0 mm DV to bregma, at a 0°angle) with 0.5–0.6 μL bilaterally (flow rate: 0.05–0.06uL/min). The syringe remained in place for an additional 10 min to reduce backflow. Optical fibers (length, 6 mm; 0.22 NA; 200 nm core from RWD life sciences, Dover, DE) targeted the ACC (+1.0 mm AP, ± 3.0 mm ML, −3.0 mm DV, at a 15° angle) and anchored to the skull with cement (C&B-Metabond, Parkell, Brentwood, NY; Ortho Acrylic, Lang Dental, Wheeling, IL). Rats were administered an analgesic (Meloxicam, 1mg/Kg or Flunixin, 1–2 mg/Kg) subcutaneously, and triple antibiotic was applied around the surgical incision. Rats recovered for a minimum of 3 weeks prior to behavioral training to allow for sufficient viral expression (6 weeks before first laser test).

### 2.3 Behavior

For solitary PMA, rats were trained as previously described (Bravo-Rivera et al., 2014; Diehl et al., 2018). Briefly, rats were conditioned with a pure tone (30 s, 4 kHz, 75 dB) co-terminating with a scrambled footshock (2 s, 0.4 mA). The inter-trial interval (ITI) averaged 3 min. An acrylic square platform (14.0 cm each side, 0.33 cm tall) located in the opposite corner of the food dish allowed rats to escape shock. The platform was fixed to the floor and present during all training stages. Rats were trained for 10 days with nine tone-shock pairings per day. A VI-30 schedule was maintained across all training and test sessions.

For social partner PMA, rats were conditioned with the same tone, footshock, and ITI parameters, but with another rat located opposite from a perforated plexiglass barrier in a reconfigured shuttle-box operant chamber which allowed rats access to their own lever, food dish, and platform. Learner Rats were paired with partners that were either previously trained in PMA (Trained Partners) or were naïve to the task (Learner Rat) prior to social partner PMA training. Partners were same-sex and non-cagemates. Rats underwent PMA training with their partner across all daily sessions. After 10 days of social partner PMA, rats underwent an additional session in the absence of their partner on Day 11 (9 tone-shock presentations).

Following 10 days of PMA training, rats in the solitary optogenetic group underwent a test of avoidance expression (2 tones presented without shock) (for details, see Diehl et al., 2018; Diehl et al., 2020). Rats in the social partner optogenetic group underwent two expression tests, one in the presence and one in the absence of their partner. Expression tests were counterbalanced to prevent any order effects.

### 2.4 Viruses

The adeno-associated viruses (AAVs; serotype 5) were obtained from the University of North Carolina Vector Core (Chapel Hill, NC). Viral titers were 4 × 10^12^ particles/mL for archaerhodopsin (AAV5:CaMKIIα::eArchT3.0-eYFP), and 3 × 10^12^ particles/mL for enhanced yellow fluorescent protein (eYFP) control (AAV5:CaMKIIα::eYFP). Rats expressing eYFP control were used to control for changes due to laser-induced heating of tissue (Stujenske et al., 2015). The CaMKIIα promoter was used to enable transgene expression favoring pyramidal neurons (Liu and Jones, 1996) in cortical regions (Jones et al., 1994; Van der Oever et al., 2013; Warthen et al., 2016). Viruses were housed in a −80°C freezer until the day of infusion.

### 2.5 Laser delivery

ACC neurons were bilaterally illuminated using a DPSS green laser (532 nm, constant, 10–12 mW at the optical fiber tip; OptoEngine, Midvale, UT). The laser was activated at tone onset during Tone 1 of the test and persisted throughout the 30 s tone presentation. Laser light passed through a shutter/coupler (200 nm, SRS, Stanford, CA), patch cord (200 nm core, ThorLabs, Newton NJ), rotary joint (200 nm core, 1×2, Doric Lenses, Quebec City, Canada), dual patch cord (0.22 NA, 200 nm core, ThorLabs or RWD life sciences), and optical fibers targeting ACC. Rats were familiarized with dummy patch cords prior to tests.

### 2.6 Open field task

Locomotion was automatically assessed (ANY-Maze, Stoelting Co, Wood Dale, IL) in an open field arena (90 cm diameter) during 30 s laser off and 30 s laser on time periods. A 6 min acclimation period preceded laser illumination. Speed and distance traveled were used to assess locomotion, and time in the center was used to assess anxiety.

### 2.7 Pressing test

Lever-pressing was assessed with a VI-30 schedule and began with a 60 s acclimation period, followed by 7 Laser On (30 s) trials and 6 Laser Off (60 s) intervals. The number of lever presses was compared during Laser Off and Laser On periods within subjects using a paired-t-test.

### 2.8 Histology

After experiments, rats were deeply anesthetized with sodium pentobarbital (450 mg/kg i.p.) and transcardially perfused with 0.9 % saline followed by a 10 % formalin solution. Brains were removed and stored in 30 % sucrose for cryoprotection for at least 72 h before sectioning. Coronal sections were cut (40 μm), mounted on slides and analyzed for viral expression and optical fiber placement.

### 2.9 Data Collection and Analysis

Behavior was recorded with digital video cameras and quantified using ANY-Maze software (Stoelting, Wood Dale, IL). The number of shocks avoided was calculated as the rat spending at least 1.75 s on the platform during the 2 s shock period of each tone presentation. Shock reactivity was calculated using the maximum speed of each rat during exposure to the first shock on Day 1 of PMA. Darting bouts, which are characterized by a rapid movement across the chamber reaching a velocity greater than 23.5 cm/s (Gruene et al., 2015) and lasting a maximum of 1 second, were calculated during the tone periods outside of the shock (i.e., the first 28 sec of each tone presentation).

Multilevel regressions were performed to assess significant differences in several behaviors observed during PMA. Multilevel regressions are powerful statistical models that can account for multiple behaviors (i.e. avoidance, freezing, pressing) that may be distributed in a binomial or Poisson fashion, and account for fixed and random effects (Bolker, 2015). Multilevel regressions have the ability to report the probability that a behavior is likely to occur based on observations of the data (Sommet & Morselli, 2017) and can better accommodate potential issues with complex datasets compared to ANOVAs (Bolker, 2015). For each behavior of interest, we used training day (10), group type (social/solitary), and sex as fixed variables and individual variation and trial (9 tone-shock presentations) as random effects for each regression model. In addition, the behaviors that were measured based on a percentage of the 30 sec tone period (i.e., time on platform and freezing) were entered into the models as proportion data and assigned a weight value. For percentage of time spent on the platform and time spent freezing during the tone (with the maximum time, 30 seconds, included as a weight value for the proportion data), multilevel binomial logistic regressions were performed to account for individual differences and proportion data being bounded by zero and one (Bolker, 2015). For number of shocks avoided, number of darting bouts during the tone, and number of ITI presses (30 s prior to tone onset), multilevel negative binomial regressions were performed to account for individual differences and positively skewed count data. To investigate effects of Partner Type (Learner Rat vs. Trained Partner), additional models were run only on the social PMA data, with Partner Type added as a predictor variable instead of Group Type. Parameter estimates for each of these models are available in Supplementary Tables S1–S10.

**Figure 1.**
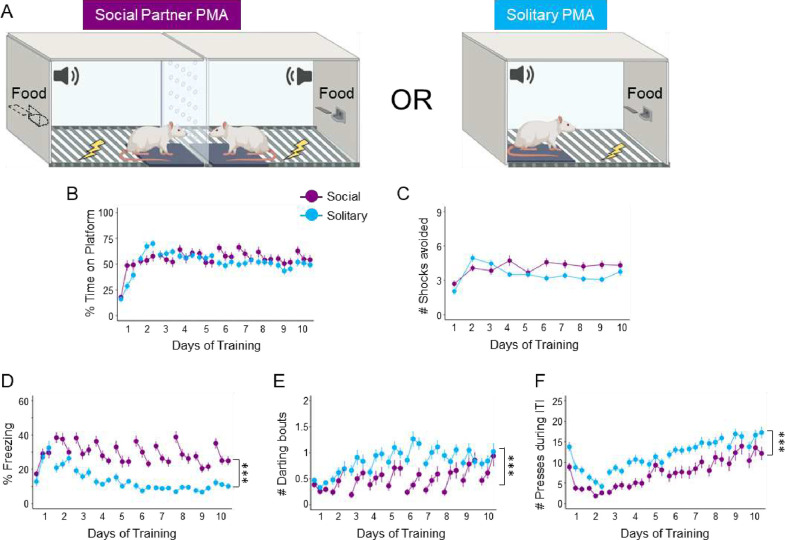
Platform-mediated avoidance (PMA) under social conditions increases freezing and decreases pressing during the intertrial interval (ITI) compared to PMA under solitary conditions. **A.** Schematic of PMA under social (left, purple, n=42) and solitary (blue, n=59) conditions. **B.** Percentage of time spent on the platform during the tone, **C.** Number of shocks avoided per day, **D.** Percentage of time freezing during the tone, **E.** Number of darting bouts during the tone, and **F.** Number of lever presses during the ITI. There was no significant difference in time on platform (z = −1.252, p=0.211) or number of shocks avoided (z=−0.552, p=0.581). There was a significant increase in freezing (z=4.068, p<0.001) and a significant decrease in darting (*z* = −4.786, *p* < 0.001) and ITI pressing (z=−6.034, p<0.001) in rats trained under social compared to solitary conditions. Data reported are main effects of the regression models (see Supplementary tables S1–S5). Data are shown across 10 days of training (trials shown in blocks of 3) and as mean ± SEM; ***p<0.001.

**Figure 2.**
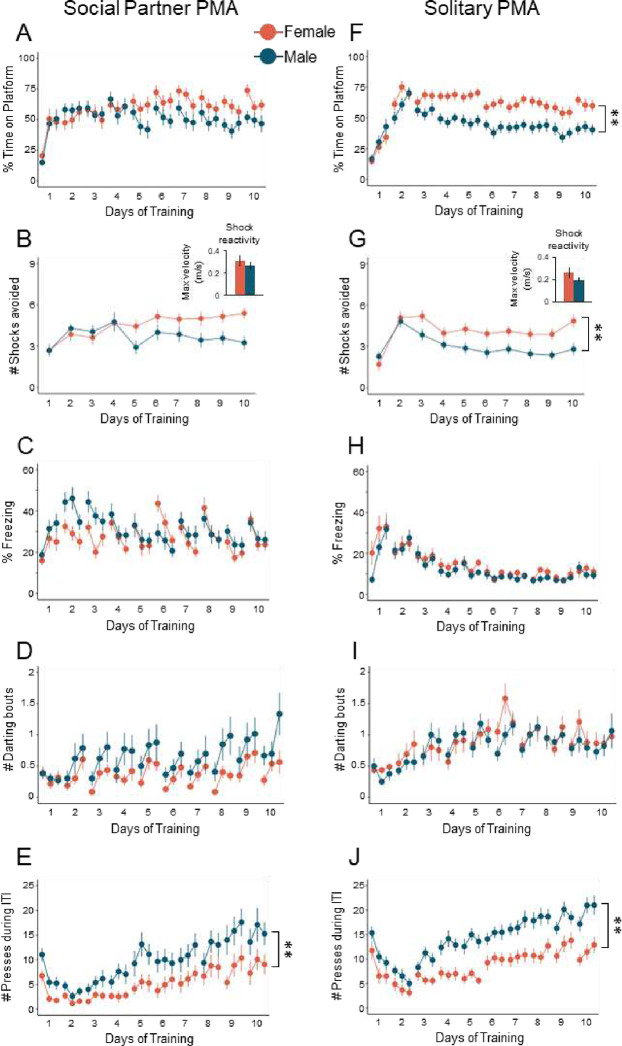
PMA under social conditions reduces behavioral sex differences that are observed during PMA under solitary conditions. **A.** Percentage of time on platform during the tone, **B.** Number of shocks avoided and shock reactivity (inset), as measured by the maximum velocity of each rat during Tone 1 on the first day of training, **C.** Percentage of time freezing during the tone, **D.** Number of darting bouts during the tone, and **E.** Number of lever presses during the ITI in female (n=21, salmon) and male (n=21, teal) rats trained under social conditions. During social partner PMA training, females pressed significantly less than males (z=−3.112, p=0.002), but there were no sex differences in time on platform (z=1.308, p=0.191), number of shocks avoided (z=1.590, p=0.112), freezing (z=−0.673, p=0.501), or darting (z=−0.886, p=0.375). **F.** Percentage of time on platform during the tone, **G.** Number of shocks avoided and shock reactivity (inset), **H.** Percentage of time freezing during the tone, **I.** Number of darting bouts during the tone, and **J.** Number of presses during the ITI in female (n=27, salmon) and male (n=32, teal) rats trained under solitary conditions. During solitary PMA training, females spent significantly more time on the platform (z=3.174, p=0.002), avoided significantly more shocks (z=2.949, p=0.003), and pressed significantly less (z=−3.044, p=0.002) compared to males. Data reported are post-hoc Tukey tests on the regression models. Data are shown across 10 days of training (trials shown in blocks of 3) and as mean ± SEM; **p<0.01.

**Figure 3.**
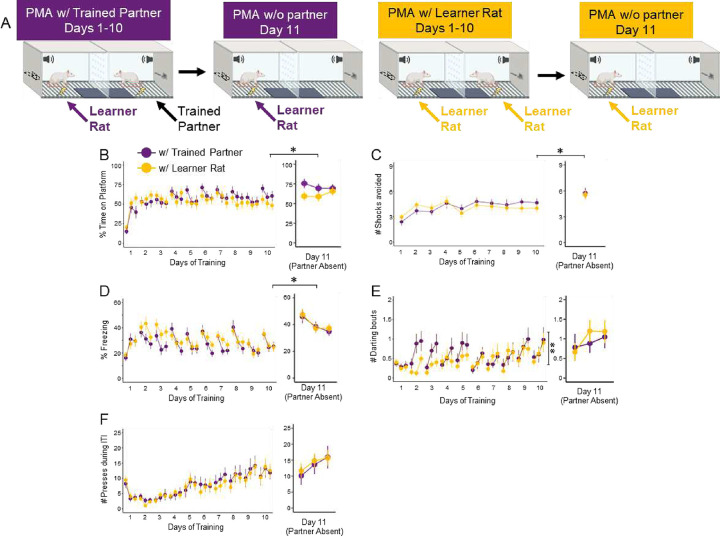
Avoidance and freezing increase in the absence of a social partner. **A.** Schematic of PMA under social conditions with a Trained Partner (n=20, purple) or another Learner Rat (n=22, yellow). **B.** Percentage of time on platform during the tone, **C.** Number of shocks avoided, **D.** Percentage of time freezing during the tone, **E.** Number of darting bouts during the tone, and **F.** Number of lever presses during the ITI across the 10 days of training (left), and on Day 11 in the absence of the partner (right). During social partner PMA training, Learner Rats with a Trained Partner darted significantly more compared to those trained with another Learner Rat (z=2.700, p=0.007). There were no differences in avoidance (time on platform: z=−1.548, p=0.122); number of shocks avoided: z=0.391, p=0.695), freezing (z=−1.168, p=0.243), or pressing (z=0.218, p=0.827) On Day 11 in the absence of the partner, Learner Rats trained with either partner type spent significantly more time on platform (with Trained Partner: z = −31.104, p < 0.001; with Learner Rat: z = −7.669, p < 0.001); avoided more shocks (with Trained Partner: z = −3.455, p < 0.001; with Learner Rat: z = −2.519, p = 0.012), and showed significantly increased freezing when trained with a Trained Partner (z = −3.921, p < 0.001). Learner Rats with a Learner Rat Partner showed significantly more darting on Day 11 compared to Day 10 (*z* = −10.019, *p* < 0.001), and those with a Trained Partner trended in the same direction (*z* = −1.953, *p* = 0.051). Data reported are main effects of the regression models (see Supplementary tables S6–S10). Day 10 vs. Day 11 comparisons are from Tukey’s post-hoc tests on the regression models. Data are shown in blocks of 3 for each day, mean ± SEM. *p<0.05, **p<0.01.

All analyses for experiments in Figures 1–3 were conducted in R (version 4.2.1), using the lme4 library, version 1.1–30 (Bates et al., 2014). The emmeans library, version 1.8.0 (Lenth et al., 2022) was used to calculate post-hoc Tukey tests and estimated marginal means from each model (all reported means are model estimates). For analyzing effects of optogenetic manipulations on behavior, repeated-measures ANOVA, followed by post-hoc Tukey analyses, or Student’s two-tailed t tests were used where appropriate using Prism (Graphpad, La Jolla, CA), or JMP (SAS, Cary, NC) software. Some of the data points in the solitary PMA group were lost for behavioral measures during acquisition, and latency to avoid and suppression of bar pressing during the laser test. Aspects of operant box schematics were created with Biorender.com.

## 3. Results

### 3.1 Platform-mediated avoidance (PMA) under social conditions increases freezing and decreases pressing compared to PMA under solitary conditions.

Previous studies of PMA under solitary conditions have shown that freezing during the tone decreases and avoidance increases as rats progress through training (Bravo-Rivera et al., 2014; Martínez-Rivera et al., 2020). To investigate how the presence of another rat can affect avoidance acquisition, several behaviors across PMA training were compared under social or solitary conditions. One group of rats was trained in social partner PMA, in which rats underwent training simultaneously with another rat (Figure 1A, left), and another group of rats was trained in solitary PMA, in which they learned avoidance alone (Figure 1A, right). Both groups underwent training for 10 days, as previously described (Bravo-Rivera et al., 2014; Diehl et al., 2018). Across 10 days of training, both social (purple) and solitary (blue) groups showed similar levels of avoidance, as measured by the percentage of time spent on the platform during the tone (Figure 1B) and the average number of shocks avoided on each day (Figure 1C). There was no significant effect of Group Type for time on platform (social vs. solitary; *z* = −1.252, *p* = 0.211) or number of shocks avoided (*z* = −0.552, *p* = 0.581).

Interestingly, rats trained under social conditions showed greater freezing compared to rats trained under solitary conditions (Figure 1D). A multilevel regression showed a significant effect of Group Type predicting time freezing (*z* = 4.068, *p* < 0.001), with social rats freezing more than solitary rats. In addition, solitary rats exhibited significantly more darting bouts during the tone (Figure 1E, *z* = −4.786, *p* < 0.001) and pressed significantly more than social rats (Figure 1F, *z* = −6.304, *p* < 0.001). Therefore, PMA training under social conditions enhanced freezing responses while decreasing food-seeking and darting, with little effect on avoidance, compared to PMA training under solitary conditions.

### 3.2 Solitary PMA reveals behavioral sex differences that are not present during social partner PMA.

The majority of PMA studies have used only male rats (Bravo-Rivera et al., 2014; Bravo-Rivera et al., 2015; Diehl et al., 2018; Diehl et al., 2020; Martínez-Rivera et al., 2018; Martínez-Rivera et al., 2020; Rodriguez-Romaguera et al., 2016). However, recent studies have included both sexes (Gabriel et al., 2022; Gongwer et al., 2023; Halcomb et al., 2023). To investigate sex differences in PMA under social and solitary conditions, we included both male and female rats in all experiments. Data in Figure 2A–H is the same data as in Figure 1 but separated by males (teal) and females (salmon). Post-hoc Tukey tests on the previous regression models showed that males and females under social conditions showed no significant differences in avoidance (Figure 2A; *z* = 1.308, *p* = 0.191), number of shocks avoided (Figure 2B; *z* = 1.590, *p* = 0.112), freezing (Figure 2C; *z* = −0.673, *p* = 0.501), or darting (Figure 2D; *z* = −0.886, *p* = 0.375). However, males showed significantly increased pressing compared to females (Figure 2E; *z* = −3.112, *p* = 0.002).

During solitary PMA, females avoided significantly more than males, as measured by time on platform (Figure 2F; *z* = 3.174, *p* = 0.002;) and number of shocks avoided (Figure 2G; *z* = 2.949, *p* = 0.003). This effect was not due to shock reactivity, as measured by maximum velocity during the first shock (Figure 2G, inset, t-test, t_(47)_=1.465, *p*=0.150). Post-hoc Tukey tests on the regression model showed no significant differences between solitary males and females in freezing (Figure 2H; *z* = 1.903, *p* = 0.057) or darting (Figure 2I; *z* = 0.401, *p* = 0.688), but males pressed more than females (Figure 2J, *z* = −3.044, *p* = 0.002). Altogether, these results suggest that any sex differences present during solitary PMA are suppressed during social partner PMA.

We next investigated whether there were any effects of training type within males or females using contrast tests on the previous regression models. Post-hoc Tukey tests identified significantly greater freezing in social compared to solitary females (*z* = 4.77, *p* < 0.001) and greater freezing in social compared to solitary males (Supplementary Figure 1C and H, *z* = 7.449, *p* < 0.001). We also observed significantly greater darting in solitary compared to social females (*z* = −4.423, *p* < 0.001) and greater darting in solitary compared to social males (*z* = −3.179, *p* = 0.002; Supplementary Figure 1D and E). Finally, significantly greater pressing was found in solitary compared to social females (*z* = −4.069, *p* < 0.001), and males (Supplementary Figure 1E and J, *z* = −3.623, *p* < 0.001). Collectively, social partner PMA training enhanced freezing and reduced darting and food seeking, regardless of sex.

### 3.3 Previous PMA experience of a social partner does not affect acquisition but alters avoidance in the absence of the partner.

We were also interested in whether prior experience of a social partner affects avoidance. Therefore, all rats that underwent the social partner PMA task were paired with either a same-sex non-cagemate partner that had previously undergone PMA (Learner Rat with a Trained Partner) or a same-sex non-cagemate partner that was also naïve to the task at the start of PMA (Learner Rat with another Learner Rat; Figure 3A). We compared the same behaviors (time on platform, number of shocks avoided, freezing, darting, and pressing) across the 10 days of training in Learner Rats trained with a Trained Partner (purple) or Learner Rats trained with another Learner Rat (yellow; data for both of these Learner Rats were combined for analysis). Our regression models (detailed in Methods) found no significant effect of Partner Type for time on platform (Figure 3B; *z* = −1.548, *p* = 0.122), number of shocks avoided (Figure 3C; *z* = 0.391, *p* = 0.695), freezing (Figure 3D; *z* = −1.168, *p* = 0.243), or pressing (Figure 3F; *z* = 0.218, *p* = 0.827). There was a significant main effect of Partner Type on darting (Figure 3E; *z* = 2.700, *p* = 0.007), with more darting bouts in Learner Rats with a Trained Partner. Overall, rats learn social partner PMA at a similar rate, regardless of the partner’s level of experience.

We were next interested in whether the partner’s absence would alter behaviors after PMA training (see Day 11 in Figure 3A). We compared the same behaviors in the presence of their partner (Day 10) versus in the absence of their partner (Day 11) using contrast comparisons on the previous regression models. Learner Rats with either Partner type spent significantly more time on the platform on Day 11 than Day 10 (Figure 3B, right, Trained Partner *z* = −31.104, *p* < 0.001; Learner Rat *z* = −7.669, *p* < 0.001) and avoided more shocks on Day 11 than Day 10 (Figure 3C, right, Trained Partner *z* = −3.455, *p* < 0.001; Learner Rat *z* = −2.519, *p* = 0.012). Learner Rats with a Trained Partner spent significantly more time freezing on Day 11 than Day 10 (Figure 3D, right, *z* = −3.921, *p* < 0.001). Learner Rats trained with another Learner Rat darted more on Day 11 than Day 10 (Figure 3E, right, Learner Rat *z* = −10.019, *p* < 0.001), whereas Learner Rats with a Trained Partner trended in the same direction (*z* = −1.953, *p* = 0.051). Finally, post-hoc comparisons on the regression model reported small significant differences in ITI pressing between Day 10 and 11 for Learner Rats with either Partner Type (Figure 3F, right, Trained Partner *z* = −15.022, *p* < 0.001; Learner Rat Partner *z* = −17.218, *p* < .001), with more pressing on Day 11 regardless of Partner Type.

### 3.4 Photoinactivation of ACC impairs avoidance under social conditions.

**Figure 4.**
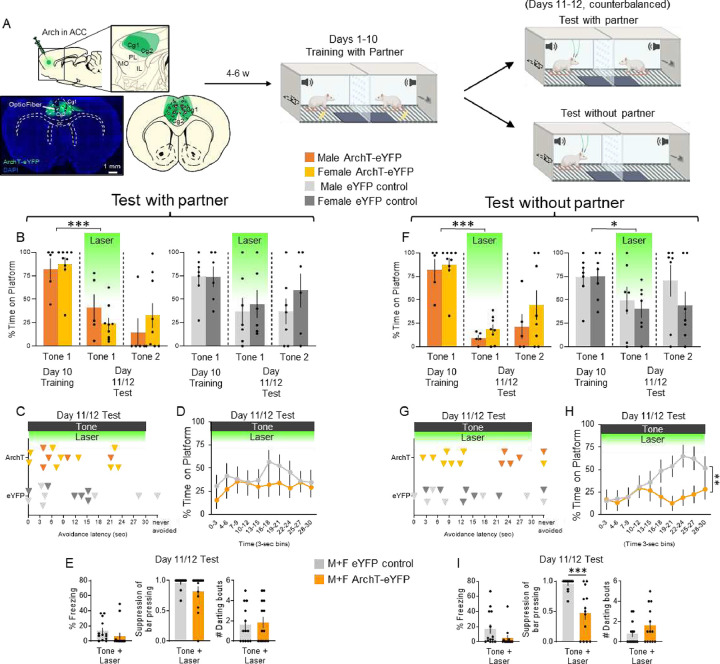
Photoinactivation of ACC projection neurons impairs avoidance under social conditions regardless of partner presence. **A.** Schematic of virus infusion, location of min/max expression of AAV, followed by avoidance training and tests. At Test w/ partner and w/o partner (Days 11/12), 532nm light was delivered to ACC during the entire 30-second tone presentation (Tone 1). **B.** Percentage of time on platform at Training (Day 10, Tone 1) and Test (Days 11/12, Tone 1 with laser ON and Tone 2 with laser OFF) for ArchT-eYFP (n=14, orange) and eYFP control rats (n=13, grey) when the partner was present. Avoidance decreased in ArchT-eYFP rats (repeated measures ANOVA, main effect of trial (F_(2,38)_=13.88, p<0.001) and interaction between trial and AAV (F_(2,38)_=3.70, p=0.034); post hoc Tukey test revealed a significant decrease between Tone 1 of Day 10 vs. Day 11 in ArchT-eYFP (p=0.002), but not in eYFP controls (p=0.478)). **C.** Latency of avoidance for each rat during Test w/ partner (Tone 1 at Test; Male ArchT, light orange, Female ArchT, dark orange; Male eYFP control, light grey; Female eYFP control, dark grey). **D.** Percentage of time on platform in 3 s bins (Tone 1 at Test) revealed no effect of photoinactivating ACC neurons in the presence of the partner (repeated measures ANOVA, post hoc Tukey, F_(9,126)_= 0.891, p=0.535). **E.** Percentage of freezing (left), suppression of bar pressing (middle), and number of darting bouts (right) during the Tone+Laser trial. **F.** Percentage time on platform at Training (Day 10, Tone 1) and Test (Day 11, Tone 1 with laser ON and Tone 2 with laser OFF) for ArchT-eYFP (n=14, orange) and eYFP control rats (n=14, grey) when the partner was absent. Avoidance decreased in ArchT-eYFP rats (repeated measures ANOVA, main effect of trial (F_(2,40)_=21.79, p<.001) and interaction between trial and AAV (F(2,40)=4.21, p=.022); post hoc Tukey test revealed a significant decrease between Tone 1 of Day 10 vs. Day 11 in ArchT-eYFP (p<0.001), but also in eYFP controls (p=0.008)). **G.** Latency of avoidance for each rat during Test w/o partner (Tone 1 at Test; Male ArchT, light orange, Female ArchT, dark orange; Male eYFP control, light grey; Female eYFP control, dark grey). **H.** Percentage of time on platform in 3 s bins (Tone 1 at Test) revealed a significant reduction in avoidance in ArchT-eYFP rats compared to eYFP controls in the absence of the partner (repeated measures ANOVA, post hoc Tukey, *F*(_9,135)_=3.09, p=0.002). **I.** Percentage of freezing (left), suppression of bar pressing (middle), and number of darting bouts (right) during the Tone+Laser trial revealed a trend toward less freezing in ArchT-eYFP rats (t_(20)_=2.036, p=0.0552) and significantly lower suppression of bar pressing in ArchT-eYFP rats (t_(20)_=3.225, p=0.0042). All data are shown as mean ± SEM; #p<0.06, *p<0.05, **p<0.01, ***p<0.001.

Previous studies have linked ACC activity with social learning (Apps et al., 2016; Burgos-Robles et al., 2019; Debiec & Olsson, 2017). We therefore reasoned that ACC activity would be necessary for social partner PMA. To assess this, we used an optogenetic approach to test if inactivation of ACC neurons would impair avoidance under social conditions. Following viral infusion of Archaerhodopsin (ArchT-eYFP) targeting ACC glutamatergic projection neurons and surgical placement of optical probes, the virus was allowed to express for 4–5 weeks after which rats were trained in PMA over 10 days as previously described (Diehl et al., 2018; Diehl et al., 2020), but in the presence of a social partner (Fig 4A). Histological analysis confirmed that expression of ArchT-eYFP was largely confined to the ACC (including anatomical areas Cg1 and Cg2) with minimal spread to secondary motor cortex (M2; lateral to Cg1), or prelimbic cortex (PL; anterior to Cg2 and ventral to Cg1) (Figure 4A, bottom left).

Following 10 days of social partner PMA (Figure 4A, middle), Learner rats underwent two avoidance expression tests: one in the presence of their partner and another in the absence of their partner (Figure 4A, top and bottom right, respectively). Each test included two tone presentations with no shock, and the order of each test was counterbalanced across rat pairs. Green laser was presented concurrently with the first 30 s tone (Laser ON trial), but not during the second tone (Laser OFF trial). 2-way repeated measures ANOVAs were used to compare the percentage of time spent on the platform in ArchT-eYFP and eYFP controls during Tone 1 on the last day of training, and Tones 1 (Laser ON) and 2 (Laser OFF) of the test sessions. When the partner was present, there was a significant main effect of trial (F_(2,38)_=13.88, *p*<0.001) and interaction between trial and AAV (F_(2,38)_=3.70, *p*=0.034), but not a significant main effect of AAV (F_(1,19)_=1.81, *p*=0.194; data collapsed across partner type and sex). Post-hoc Tukey tests revealed a significant decrease in time on platform between Tone 1 on the last day of training and Tone 1 of test (Laser ON) in the ArchT-eYFP group (Figure 4B; *p*=0.002), but not in the eYFP control group (*p*=0.478). When comparing avoidance latency across the tone + laser trial between ArchT-eYFP and eYFP control rats, there was no significant difference (Figure 4C; t_(19)_=1.122, *p*=0.276), nor across the timecourse of avoidance, as measured in 3 s bins of the tone period (Figure 4D; repeated measures ANOVA: F_(9,126)_= 0.891, *p*=0.535). Finally, photoinactivation had no effect on freezing (t_(25)_=1.192, *p*=0.245) suppression of bar pressing (t_(25)_=1.588, *p*=0.125), nor on darting (t_(25)_=0.326) in the presence of the partner (Figure 4E).

In the absence of the partner, there was a significant main effect of trial (F_(2,40)_=21.79, *p*<0.001) and interaction between trial and AAV (F_(2,40)_=4.21, *p*=0.022), but not a significant main effect of AAV (F_(1,20)_=1.01, *p*=0.328). Post-hoc Tukey tests revealed a significant decrease in time spent on the platform between Tone 1 on the last day of training and Tone 1 of test (Laser ON) in the ArchT-eYFP group (Figure 4F; *p*<0.001), but also in the eYFP control group *(p*=0.008). Photoinactivation had no effect on avoidance latency (Figure 4G, t_(20)_=0.539, *p*=0.596), but when comparing the timecourse of avoidance between ArchT-eYFP and eYFP control rats, photoinactivation significantly reduced avoidance throughout the tone (Figure 4H, F_(9,135)_= 3.09, *p*=0.002)). Photoinactivation had no effect on freezing (t_(25)_=1.654, *p*=0.111), a significant effect on suppression of bar pressing (t_(25)_=4.473, *p*=0.001), but no effect on darting (t_(25)_=1.447, *p*=0.160) in absence of the partner (Figure 4I). Finally, to determine whether differences in social partner PMA training might account for effects observed during photoinactivation, we compared time on platform, number of shocks avoided, freezing, and ITI pressing during the 10 days of training. eYFP control rats spent more time on the platform than ArchT-eYFP rats (F_(1,14)_=6.04, p=0.028), but this effect was not seen in Tukey’s post hoc comparisons across days. There were no significant differences in number of shocks avoided (F_(1,14)_=2.73, p=0.121), freezing (F_(1,14)_=0.006, p=0.941) or lever-pressing during the ITI (F_(1,14)_=0.624, p=0.443) between ArchT-eYFP and eYFP control rats (Supplementary Figure 2A).

### 3.5 Photoinactivation of ACC delays avoidance under solitary conditions in male rats but blocks avoidance in female rats.

**Figure 5.**
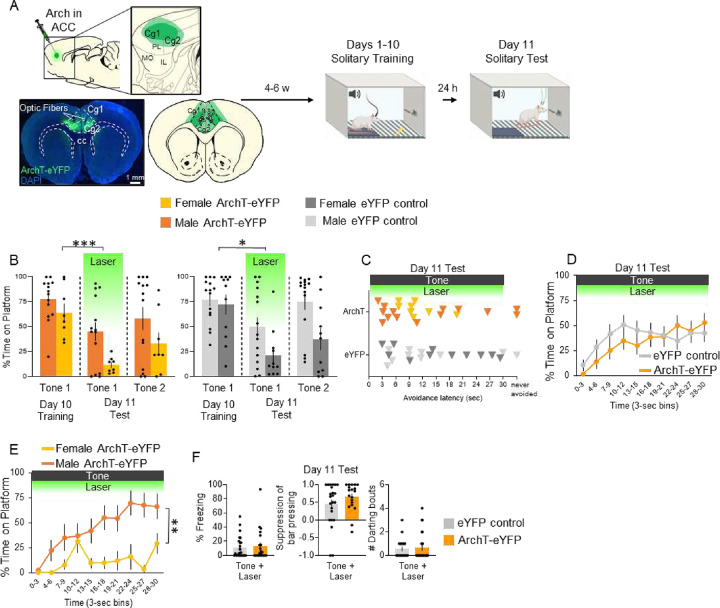
Photoinactivation of ACC projection neurons delays avoidance expression in male rats but blocks avoidance expression in female rats under solitary conditions. **A.** Schematic of virus infusion, location of min/max expression of AAV, followed by avoidance training and test. At Test, 532nm light was delivered to ACC during the entire 30-second tone presentation (Tone 1). **B.** Percentage of time on platform at Training (Day 10, Tone 1) and Test (Day 11, Tone 1 with laser ON and Tone 2 with laser OFF) for ArchT-eYFP (n=22, left, orange) and eYFP control rats (n=26, right, grey). Avoidance was impaired in ArchT-eYFP rats (repeated measures ANOVA, main effect of trial (F_(2,70)_=18.49, p<0.001); post hoc Tukey test revealed a significant decrease between Tone 1 of Day 10 vs. Day 11 in ArchT-eYFP (p<0.001), but also in eYFP controls (p=0.004)). **C.** Latency of avoidance for each rat (Tone 1 at Test). There was no significant difference between ArchT-eYFP and eYFP control rats (t_(41)_=0.815, p=0.420). **D.** Percentage of time on platform in 3 s bins (Tone 1 at Test) revealed no significant differences between groups (repeated measures ANOVA, post hoc Tukey, F_(9,279)_=1.25, p=0.265). **E.** Percentage of time on platform in 3 s bins (Tone 1 at Test) revealed a significant delay in avoidance when silencing ArchT-eYFP neurons in male rats (light orange) while blocking avoidance in female rats (dark orange; repeated measures ANOVA F_(9,162)_=3.76, p<0.001, post hoc Tukeys, all p’s<0.001 15–30 sec). **F.** Percentage of freezing (left), suppression of bar pressing (middle), and number of darting bouts (right) during the Tone+Laser trial. Data shown as mean ± SEM;*p<0.05; **p<0.01; ***p<0.001.

To determine whether activity in ACC may also be necessary for avoidance under solitary conditions, we photoinactivated these neurons during an expression test following solitary PMA. Similar to the experiment in Figure 4, rats were infused with ArchT-eYFP or eYFP control virus and subsequently underwent solitary PMA training (Figure 5A). A 2-way repeated measures ANOVA comparing the percentage of time spent on the platform in ArchT-eYFP and eYFP controls during Tone 1 of the last day of training, and Tones 1 (Laser ON) and 2 (Laser OFF) of the test session showed a significant main effect of trial (F_(2,70)_=18.49, *p*<0.001), but no main effect of AAV (F_(1,35)_=2.83, *p*=0.102), or their interaction (F_(2,70)_=0.555, *p*=0.577). Post-hoc Tukey tests revealed a significant decrease in time spent on the platform between Tone 1 on the last day of training and Tone 1 of test (Laser ON) in the ArchT-eYFP group (Figure 5B; *p*<0.001) but also in the eYFP control group (*p*=0.004). There was no effect on avoidance latency (Figure 5C; t_(41)_=0.815, *p*=0.420), nor on the timecourse of avoidance (Figure 5D; repeated measures ANOVA, F_(9,279)_=1.25, *p*=0.265).

Interestingly, when examining sex differences among the ArchT-eYFP groups, males showed a delay in avoidance compared to female rats that showed a near complete impairment of avoidance across the 30 s tone (comparing light and dark orange lines across both graphs in Figure 5E; repeated measures ANOVA, *F*_(9,162)_=3.76, *p<*0.001, post hoc Tukeys, all p’s<0.001 after 15 s). Thus, photoinactivation of ACC somata during the tone blocked avoidance in females, but delayed avoidance in males. Photoinactivation had no effect on freezing (Figure 5F; t_(46)_=0.403, *p*=0.689) or suppression of bar pressing (t_(40)_=1.268, *p*=0.212), or darting (t_(46)_=0.369, *p*=0.714). Finally, photoinactivation had no effect on spontaneous bar-pressing between a subset of ArchT-eYFP and eYFP controls (t-test, t_(24)_=0.343, *p*=0.738; ArchT-eYFP n=11, M=4.7, eYFP n=13, M=4.8). Photoinactivation also had no effect on locomotion, as measured by distance traveled in an open field (t_(41)_ = −1.22, *p*=0.232; ArchT-eYFP n=17, M=1.1 m, eYFP n=24,M=0.97 m), nor on anxiety levels, as both groups spent similar amounts of time in the center of the open field (t_(41)_ = −1.35, *p*=0.184; ArchT-eYFP n=17, M=3.3 s, eYFP n=24, M=2.0 s). Finally, during solitary PMA training, there were no significant differences in time on platform (F_(1,20)_=0.447, p=0.511), number of shocks avoided (F_(1,20)_=0.005, p=0.944), freezing (F_(1,20)_=1.758, p=0.200), or lever-pressing during the ITI (F_(1,20)_=0.960, p=0.339), between ArchT-eYFP and eYFP control rats (Supplementary Figure 2B).

## 4. Discussion

We have developed a behavioral task to assess the acquisition of PMA under social conditions and compared learning between male and female rats trained in PMA under social or solitary conditions. We found that females avoided more than males, whereas males pressed more for sucrose reward than females during PMA training. Sex differences were more prominent in solitary vs. social rats. Thus, a social context of aversive learning appears to suppress sex differences, causing female rats to “act more like” male rats. We also found that, regardless of sex, freezing was enhanced while darting and food seeking were suppressed under social compared to solitary conditions. In addition, when rats underwent social partner PMA, the prior experience of their partner had little effect on behavior during training. However, removing the partner increased avoidance and freezing regardless of partner type. We next found that ACC activity was necessary for the expression of PMA in rats that learned under either social or solitary conditions but in different ways. In social partner PMA, ACC activity was necessary for the expression of avoidance both in the presence and absence of the partner. In solitary PMA, ACC activity modulated avoidance expression in a sex-dependent manner, such that photo-inactivating ACC had a milder effect of delaying avoidance in males, but a stronger effect of blocking avoidance in females. Below, we highlight some unexpected findings from our study along with possible explanations and future research directions.

The surprising finding that freezing increased during social partner PMA compared to solitary PMA (Figure 1C) suggests that Learner Rats associated their partner with danger. This agrees with studies showing that rats can associate another rat with a shock US (Dawud et al., 2021). A rat that is freezing to a CS can also become a signal for danger in addition to the CS itself (Cruz et al., 2020). It is also possible that Learner Rats increased freezing during social partner PMA due to a lack of detecting any movement from their partner, similar to previous studies showing that social transmission of fear can be propagated by silence caused by the cessation of movement (Pereira et al., 2012). In the current study, it is likely that Learner Rats perceived their partner as a negative stimulus, which led to increased freezing, rather than a positive stimulus, which would have shown a buffering effect and led to less freezing. This agrees with studies showing that partner presence *during* a traumatic event does not reduce fear responses, but rather a partner present *after* a traumatic event does reduce fear responses (Gorkiewicz et al., 2023). Future studies using social partner PMA can address this question by testing if the presentation of a partner after PMA training (under solitary conditions) would reduce freezing and promote extinction of avoidance.

We also observed decreased lever pressing during the ITI under social conditions compared to solitary conditions, suggesting that Learner Rats may be less motivated to forage for food when another rat is present. This may be due to Learner Rats investigating their partner to obtain additional information about the context or task contingencies. Furthermore, rats may be using sensory cues from their partner to learn about PMA. During social learning, rodents use auditory and visual cues (Paraouty et al., 2020) as well as olfactory cues (Contestabile et al., 2021; Sanchez-Andrade & Kendrick, 2009) to gather information about the environment. In the current study, Learner Rats may observe their partner avoid, causing them to also avoid, or they may hear an alarm call emitted by their partner. Although visual attention and vocalizations were not assessed in the current study, future studies should assess whether social partner PMA relies on visual, auditory, or olfactory cues between partners.

During social partner PMA training, females avoided similarly to males (Figure 2A), which was lower than avoidance levels observed in solitary females (Supplementary Figure 2A). Previous studies have shown that female rats avoid foraging in large open areas by themselves (Zambetti et al., 2019) and overall show greater defensive responses (Blanchard et al., 1991) compared to male rats, which could explain why females spend more time on the platform than males under solitary conditions. In addition, males pressed more than females, regardless of training condition (Figure 2D & 2H), but this difference was less robust under social conditions. This might be due to the possibility that males perceive greater competition in the presence of another male (Kikusui et al., 2006). Alternatively, the observation that males show riskier behaviors than females (Ishii et al., 2018; Jolles et al., 2015; Orsini et al., 2016) could explain why males pressed more than females under both training conditions. All rats avoided more and displayed enhanced freezing when they were alone than with their partner (Figure 3B–C), which might reflect heightened vigilance or stress. Although this disagrees with studies showing that female rodents benefit more from social buffering compared to male rodents (Barnett, 2007; Ishii et al., 2016; Modlinska & Pisula, 2020), the presence of a social partner *during* PMA training did not induce a sex-dependent change in freezing. Overall, this suggests that social partner PMA can suppress sex differences that might be observed during solitary PMA.

Studies investigating social interactions and social learning have pointed to the anterior cingulate cortex (ACC) as a key region across many species (Burgos-Robles et al., 2019). Indeed, prior studies have reported neural correlates of active avoidance in rabbits performing the wheel running task (Gabriel lab studies). In addition, previous research on observational fear has demonstrated that ACC integrates information about social cues and aversive stimuli, which are mediated by connections between the ACC and basolateral amygdala (BLA) (Allsop et al., 2018).

The ACC is a highly interconnected cortical brain region, projecting densely to the PL (Conde et al., 1995; Jones et al., 2005) and BLA (Bissière et al., 2008; Cassell & Wright, 1986; Gabbott et al., 2005), making this region a possible candidate structure to regulate active avoidance. Since the ACC processes social information, these neurons may signal the PL information about the social partner since PL is a prominent regulator of avoidance in PMA (Diehl et al., 2018; Diehl et al., 2020). The ACC also comprises part of the value processing network together with the BLA, integrating information about observed emotion and social interactions (Apps et al., 2016; Burgos-Robles et al., 2019). Therefore, it is possible that ACC neurons may also signal the BLA during PMA under social conditions, thereby allowing Learner rats to use social information to avoid danger.

We found that during PMA under solitary conditions, ACC photoinactivation had a mild effect of delaying avoidance in males but had a stronger effect of blocking avoidance in females, suggesting that the ACC is recruited differentially in male and female rats. This suggests that female avoidance circuits rely more heavily on ACC, whereas male avoidance circuits may be more distributed and rely on other nodes of the avoidance circuit (Diehl et al., 2018; Diehl et al., 2020). Future studies using electrophysiological recordings could determine ACC correlates of behavior during PMA and how ACC activity differs under social vs. solitary conditions. For example, Partner Type may alter ACC activity during social partner PMA, similar to studies showing that dominance status changes neuronal activity during social interactions (Kingsbury et al., 2019).

We unexpectedly observed that eYFP controls also showed reduced avoidance during the expression test. To rule out effects of possible tissue damage due to laser heat, laser illumination was maintained between 10–12 mW (irradiance: 79–96 mW/mm^2^) at the tip of the optical fiber, a range which should not produce any phototoxic effects (Senova et al., 2017). However, it is possible that the mere introduction of photons may have altered biophysical properties within neurons (Bernard, 2020) or that rats may have experienced “optoception,” in which subjects can perceive photo-manipulation of their own brain tissue (Luis-Islas et al., 2022), which may have affected behavior in both ArchT-eYFP and eYFP control groups. Although these prior studies on issues with optogenetic illumination has largely been studied in rodents with Channelrhodopsin-infected neurons using blue light (473 nm), future studies should confirm if similar effects might also occur with ArchT-infected neurons using green light (532 nm).

Excessive avoidance is a key symptom in several neuropsychiatric illnesses including PTSD (Asmundson et al., 2004; Breslau, 2001), OCD (McGuire et al., 2012), social anxiety disorder (SAD) (Wake et al., 2021), depression (Trew, 2011), and autism spectrum disorder (ASD) (Madipakkam et al., 2017). The human homolog of the rat ACC, Brodmann areas 24a/b (Burgos-Robles et al., 2019), has been implicated in these disorders (OCD: Kosová et al., 2023; Lee et al., 2023), (SAD: Jamieson et al., 2023), (depression: Alexander et al., 2023), (ASD: Bartolotti et al., 2020). Interestingly, a meta-analysis reported that the ACC was one of seven regions with disrupted functional connectivity across different anxiety disorders (Rezaei et al., 2023). Therefore, findings of the current study are consistent with clinical studies that the ACC is an area of interest for developing treatments to resolve symptoms of these neuropsychiatric disorders.

The PMA task has previously been used as a model to study extinction-based treatment for PTSD and OCD (Martínez-Rivera et al., 2020; Rodriguez-Romaguera et al., 2016). Therefore, modifying the PMA task to include a social context could be used as an animal model of behavior to study other anxiety disorders such as SAD, in which social context is a key factor. Finally, our findings that females avoided more than males and showed a stronger impairment of avoidance when ACC was optogenetically inactivated suggest that ACC may be a key factor in understanding why females are at a greater risk of an anxiety disorder.

## Supplementary Material

Supplement 1
