# Supplementary material for: Active avoidance recruits the anterior cingulate cortex regardless of social context in male and female rats
Source: Res Sq. 2024 Jun 7:rs.3.rs-3750422. Originally published 2024 Jan 2. Preprint. [Version 2] doi: 10.21203/rs.3.rs-3750422/v2 (PMC10802695; doi:10.21203/rs.3.rs-3750422/v2)
Supplement: Supplement 1 [file NIHPPrs3750422v2-supplement-1.pdf]

Supplemental Material.

Supplementary Figure 1.

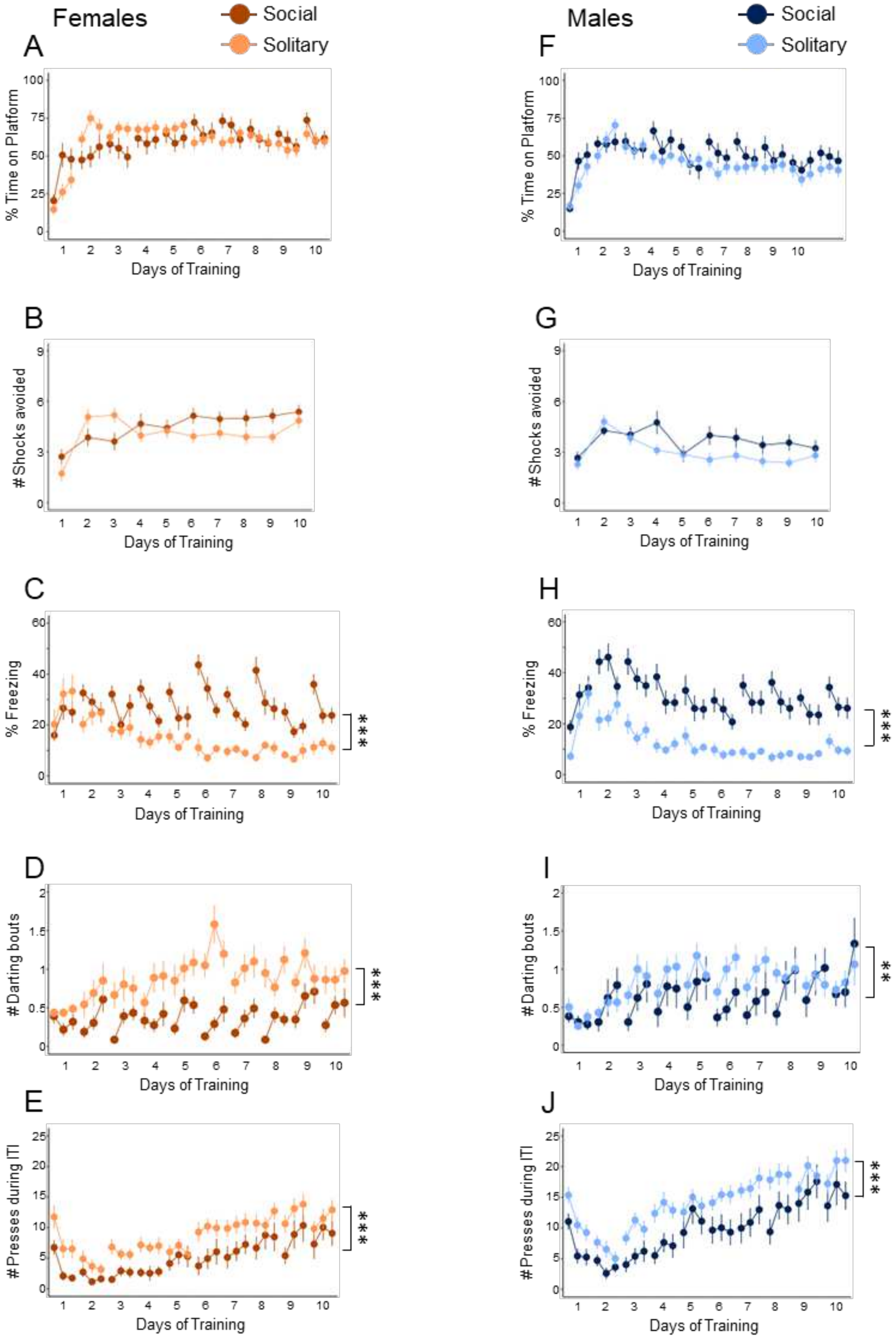

Supplementary Figure 2.

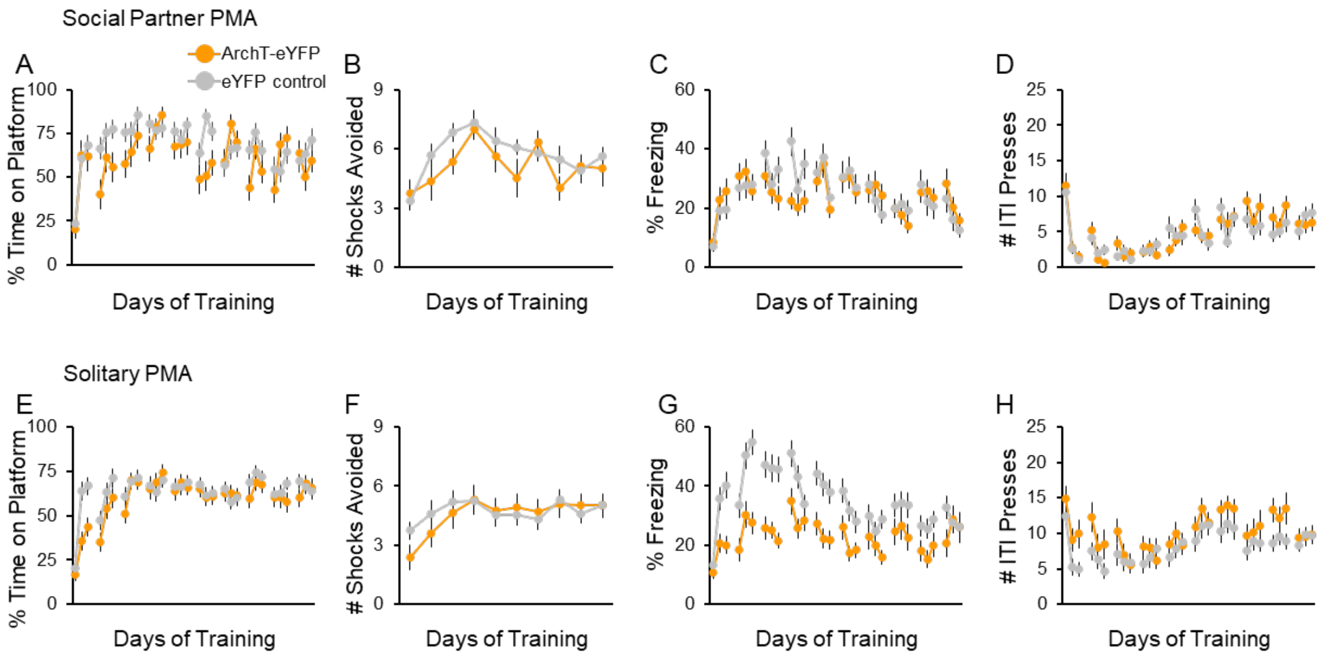

Table S1. Time on platform in social vs. solitary PMA

Fixed effects parameter estimates of the multilevel binomial logistic regression with Group Type (social vs. solitary), Sex, Trial Day, and their interactions and random intercept effects of Animal and Stimulus Number predicting the proportion of time spent on the platform. All categorical variables are effect coded. Trial Number and Stimulus Number were both rescaled to range from 0 to 1 to improve model convergence.

	B	*SE*	*z*-value	*p*-value
(Intercept)	0.126	0.092	1.366	0.172
Group Type [social]	-0.114	0.091	-1.252	0.211
Sex [female]	0.063	0.091	0.699	0.485
Trial Number	0.160	0.013	12.031	< 0.001
Group Type [social] * Sex [female]	-0.131	0.091	-1.449	0.147
Group Type [social] * Trial Number	0.257	0.013	19.321	< 0.001
Sex [female] * Trial Number	0.421	0.013	31.643	< 0.001
Group Type [social] * Sex [female] * Trial Number	0.072	0.013	5.380	< 0.001

Table S2. Number of shocks avoided in social vs. solitary PMA

Fixed effects parameter estimates of the multilevel negative binomial regression with Group Type (social vs. solo), Sex, Trial Number (1-10), and their interactions and random intercept effects of Animal and Stimulus Number predicting the number of shocks avoided. All categorical variables are effect coded.

	B	*SE*	*z*-value	*p*-value
(Intercept)	1.217	0.059	20.651	< 0.001
Group Type [social]	-0.032	0.059	-0.552	0.581
Sex [female]	-0.013	0.059	-0.229	0.819
Trial Number	1.003	0.058	0.602	0.547
Group Type [social] * Sex [female]	-0.044	0.059	-0.762	0.446
Group Type [social] * Trial Number	0.021	0.058	3.608	< 0.001
Sex [female] * Trial Number	0.030	0.058	5.209	< 0.001
Group Type [social] * Sex [female] * Trial Number	0.002	0.058	0.303	0.762

Table S3. Freezing in social vs. solitary PMA

Fixed effects parameter estimates of the multilevel binomial logistic regression with Group Type (social vs. solitary), Sex, Trial Day, and their interactions and random intercept effects of Animal and Stimulus Number predicting the proportion of time spent freezing. All categorical variables are effect coded. Trial Number and Stimulus Number were both rescaled to range from 0 to 1 to improve model convergence.

	B	*SE*	*z*-value	*p*-value
(Intercept)	-1.125	0.071	-15.785	< 0.001
Group Type [social]	0.269	0.066	4.068	< 0.001
Sex [female]	-0.019	0.066	-0.296	0.767
Trial Number	-0.785	0.016	-47.559	< 0.001
Group Type [social] * Sex [female]	-0.171	0.066	-2.580	0.009
Group Type [social] * Trial Number	0.587	0.016	36.549	< 0.001
Sex [female] * Trial Number	0.132	0.016	8.015	< 0.001
Group Type [social] * Sex [female] * Trial Number	0.111	0.016	6.705	< 0.001

Table S4 Darting in social vs. solitary PMA

Fixed effects parameter estimates of the multilevel negative binomial regression with Group Type (social vs. solitary), Sex, Trial Day, and their interactions and random intercept effects of Animal and Stimulus Number predicting the number of tone-induced darting bouts. All categorical variables are effect coded.

	B	*SE*	*z*-value	*p*-value
(Intercept)	-1.049	0.103	-10.226	< 0.001
Group Type [social]	-0.398	0.083	-4.786	< 0.001
Sex [female]	0.007	0.083	0.081	0.936
Trial Number	0.055	0.006	10.022	< 0.001
Group Type [social] * Sex [female]	-0.021	0.083	-0.257	0.797
Group Type [social] * Trial Number	-0.003	0.006	-0.472	0.637
Sex [female] * Trial Number	-0.007	0.006	-1.264	0.206
Group Type [social] * Sex [female] * Trial Number	-0.009	0.006	-1.653	0.098

Table S5. ITI pressing in social vs. solitary PMA

Fixed effects parameter estimates of the multilevel negative binomial regression with Group Type (social vs. solitary), Sex, Trial Day, and their interactions and random intercept effects of Animal and Stimulus Number predicting the number of lever presses in each ITI. All categorical variables are effect coded. Trial Number and Stimulus Number were both rescaled to range from 0 to 1 to improve model convergence.

	B	*SE*	*z*-value	*p*-value
(Intercept)	1.369	0.075	18.366	< 0.001
Group Type [social]	-0.456	0.072	-6.304	< 0.001
Sex [female]	-0.326	0.072	-4.497	< 0.001
Trial Number	1.029	0.031	33.129	< 0.001
Group Type [social] * Sex [female]	-0.053	0.072	-0.742	0.458
Group Type [social] * Trial Number	0.144	0.031	4.650	< 0.001
Sex [female] * Trial Number	0.039	0.031	1.251	0.211
Group Type [social] * Sex [female] * Trial Number	0.048	0.031	1.566	0.117

Table S6. Time on platform in Learner Rats with Trained Partners or Learner Rats

Fixed effects parameter estimates of the multilevel binomial logistic regression with Partner Type (Trained Partner vs. Learner Rat), Sex, Trial Number (1-11), and their interactions and random intercept effects of Animal and Stimulus Number predicting the proportion of time spent on the platform. Model was run on only data from social rats, with 10 days of social partner PMA and Day 11 in the absence of the partner (11 days total). All categorical variables are effect coded. Trial Number and Stimulus Number were both rescaled to range from 0 to 1.1 to improve model convergence.

	B	*SE*	*z*-value	*p*-value
(Intercept)	-0.026	0.104	-0.247	0.805
Partner Type [trained partner]	-0.158	0.102	-1.548	0.122
Sex [female]	-0.016	0.080	-0.202	0.840
Trial Number	0.501	0.018	28.038	< 0.001
Partner Type [trained partner] * Sex [female]	-0.152	0.080	-1.892	0.059
Partner Type [trained partner] * Trial Number	0.315	0.018	17.619	< 0.001
Sex [female] * Trial Number	0.415	0.018	23.235	< 0.001
Partner Type [trained partner] * Sex [female] * Trial Number	0.245	0.018	13.738	< 0.001

Table S7. Number of shocks avoided in Trained partners and Learner rats

Fixed effects parameter estimates of the multilevel binomial logistic regression with Partner Type (Trained Partner vs. Learner Rat), Sex, Trial Number (1-11), and their interactions and random intercept effects of Animal and Stimulus Number predicting the number of shocks avoided. Model was run on only data from social rats, with 10 days of partner PMA and Day 11 in the absence of the partner (11 days total). All categorical variables are effect coded.

	B	*SE*	*z*-value	*p*-value
(Intercept)	1.127	0.115	9.792	< 0.001
Partner Type [trained partner]	0.055	0.141	0.391	0.695
Sex [female]	0.022	0.115	0.189	0.850
Trial Number	0.039	0.011	3.455	< 0.001
Partner Type [trained partner] * Sex [female]	-0.103	0.141	-0.731	0.465
Partner Type [trained partner] * Trial Number	-0.013	0.015	-0.879	0.380
Sex [female] * Trial Number	0.038	0.011	3.365	< 0.001
Partner Type [trained partner] * Sex [female] * Trial Number	-0.019	0.015	-1.254	0.210

Table S8. Freezing in Trained partners and Learner rats in social PMA

Fixed effects parameter estimates of the multilevel binomial logistic regression with Partner Type (Trained Partner vs. Learner Rat), Sex, Trial Number (1-11), and their interactions and random intercept effects of Animal and Stimulus Number predicting the proportion of time spent freezing. Model was run on only data from social rats, with 10 days of partner PMA and Day 11 in the absence of the partner (11 days total). All categorical variables are effect coded. Trial Number and Stimulus Number were both rescaled to range from 0 to 1.1 to improve model convergence.

	B	*SE*	*z*-value	*p*-value
(Intercept)	-0.975	0.108	-9.062	< 0.001
Partner Type [trained partner]	-0.105	0.090	-1.168	0.243
Sex [female]	-0.206	0.077	-2.666	0.008
Trial Number	0.062	0.019	3.270	0.001
Partner Type [trained partner] * Sex [female]	-0.002	0.077	-0.032	0.974
Partner Type [trained partner] * Trial Number	0.047	0.019	2.497	0.013
Sex [female] * Trial Number	0.248	0.019	13.154	< 0.001
Partner Type [trained partner] * Sex [female] * Trial Number	0.076	0.019	4.051	< 0.001

Table S9. Darting in Trained partners and Learner rats in social PMA

Fixed effects parameter estimates of the multilevel binomial logistic regression with Partner Type (Trained Partner vs. Learner Rat), Sex, Trial Number (1-11), and their interactions and random intercept effects of Animal and Stimulus Number predicting the number tone-induced darting bouts. Model was run on only data from social rats, with 10 days of partner PMA and Day 11 in the absence of the partner (11 days total). All categorical variables are effect coded.

	B	*SE*	*z*-value	*p*-value
(Intercept)	-1.491	0.192	-7.773	< 0.001
Partner Type [trained partner]	0.413	0.153	2.700	0.007
Sex [female]	-0.080	0.153	-0.522	0.602
Trial Number	0.068	0.008	8.293	< 0.001
Partner Type [trained partner] * Sex [female]	-0.142	0.153	-0.930	0.352
Partner Type [trained partner] * Trial Number	-0.045	0.008	-5.451	< 0.001
Sex [female] * Trial Number	-0.013	0.008	-1.530	0.126
Partner Type [trained partner] * Sex [female] * Trial Number	-0.042	0.008	-5.156	< 0.001

Table S10. ITI pressing in Trained partners and Learner rats in social PMA

Fixed effects parameter estimates of the multilevel binomial logistic regression with Partner Type (Trained Partner vs. Learner Rat), Sex, Trial Number (1-11), and their interactions and random intercept effects of Animal and Stimulus Number predicting the number of lever presses in each ITI. Model was run on only data from social rats, with 10 days of partner PMA and Day 11 in the absence of the partner (11 days total). All categorical variables are effect coded. Trial Number and Stimulus Number were both rescaled to range from 0 to 1.1 to improve model convergence.

	B	*SE*	*z*-value	*p*-value
(Intercept)	0.909	0.141	6.409	< 0.001
Partner Type [trained partner]	0.030	0.139	0.218	0.827
Sex [female]	-0.297	0.122	-2.434	0.015
Trial Number	1.215	0.054	22.557	< 0.001
Partner Type [trained partner] * Sex [female]	0.087	0.122	0.712	0.476
Partner Type [trained partner] * Trial Number	-0.040	0.053	-0.749	0.454
Sex [female] * Trial Number	0.088	0.053	1.655	0.098
Partner Type [trained partner] * Sex [female] * Trial Number	-0.089	0.053	-1.673	0.094

Supplementary Figure 1. PMA under social conditions increases freezing and decreases pressing, regardless of sex. A. Percentage of time on platform during the tone, **B.** Number of shocks avoided, **C.** Percentage of freezing during the tone, **D.** Number of darting bouts during the tone, and **E.** Number of presses during the ITI in females trained under social (n=21, dark red) or solitary (n=27, orange) conditions. **F.** Percentage of time on platform during the tone, **G.** Number of shocks avoided, **H.** Percentage of freezing during the tone, **I.** Number of darting bouts during the tone, and **J.** Number of presses during the ITI in males trained under social (n=21, dark blue) or solitary (n=32, light blue) conditions. Regardless of sex, social rats spent significantly more time freezing during the tone (females: z = 4.77, p < 0.001; males: z = 7.449, p < 0.001) and darted significantly less (females: *z* = -4.423, *p* < 0.001; males: *z* = -3.179, *p* = 0.002) and pressed significantly less (females: z = -4.069, p < 0.001; males: z = -3.623, p < 0.001) compared to solitary rats. There were no significant differences in avoidance between social and solitary females (z = -0.608, p = 0.544), or between social and solitary males (z = 0.888, p = 0.375). There were no significant differences in number of shocks avoided between social and solitary females (z = 0.690, p = 0.490), or between social and solitary males (z = 1.716, p = 0.086). Data reported are post-hoc Tukey tests on the regression models. Data are shown across 10 days of training (trials shown in blocks of 3) and as mean ± SEM; **p<0.01; ***p<0.001.

Supplementary Figure 2. Acquisition of PMA under social and solitary conditions for optogenetic rat cohorts A. Percentage of time on platform during the tone, **B.** Number of shocks avoided, **C.** Percentage of freezing during the tone, and **D.** Number of lever presses during the ITI in ArchT-eYFP (n=14, orange) and eYFP controls (n=14, grey) during 10 days of PMA training under social conditions. There was a small but significant main effect of AAV in time on platform (repeated measures ANOVA, $F_{(1,14)}$=6.04, p=0.028) where eYFP controls spent more time on the platform compared to ArchT-eYFP rats; however, no Tukey's post hoc comparisons were statistically significant for any specific day of PMA training. There were no significant differences in the number of shocks avoided (repeated measures ANOVA, $F_{(1,14)}$=2.73, p=0.121), freezing (repeated measures ANOVA, $F_{(1,14)}$=0.006, p=0.941), or number of ITI presses (repeated measures ANOVA, $F_{(1,14)}$=0.624, p=0.443). **E.** Percentage of time on platform during the tone, **F.** Number of shocks avoided, **G.** Percentage of freezing during the tone, and **H.** Number of presses during the ITI in ArchT-eYFP (n=13, orange) and eYFP controls (n=20, grey) during 10 days of PMA training under solitary conditions. There were no significant differences in time on platform (repeated measures ANOVA, $F_{(1,20)}$=0.447, p=0.511), number of shocks avoided (repeated measures ANOVA, $F_{(1,20)}$=0.005, p=0.944), freezing (repeated measures ANOVA, $F_{(1,20)}$=1.758, p=0.200), or number of ITI presses (repeated measures ANOVA, $F_{(1,20)}$=0.960, p=0.339). Data shown across 10 days of training (trials shown in blocks of 3). Data are shown as mean ± SEM.
